# The Potential of Electrospinning to Enable the Realization of Energy-Autonomous Wearable Sensing Systems

**DOI:** 10.1021/acsnano.3c09077

**Published:** 2024-01-17

**Authors:** K. R. Sanjaya Dinuwan Gunawardhana, Roy B. V. B. Simorangkir, Garrett Brian McGuinness, M. Salauddin Rasel, Luz A. Magre Colorado, Sonal S. Baberwal, Tomás E. Ward, Brendan O’Flynn, Shirley M. Coyle

**Affiliations:** † School of Electronic Engineering, Dublin City University, Glasnevin D09Y074, Dublin, Ireland; ‡ Insight SFI Centre for Data Analytics, Dublin City University, Glasnevin D09Y074, Dublin, Ireland; § Tyndall National Institute, Lee Maltings Complex Dyke Parade, T12R5CP Cork, Ireland; ∥ School of Mechanical Engineering, Dublin City University, Glasnevin D09Y074, Dublin, Ireland; ⊥ School of Computing, Dublin City University, Glasnevin D09Y074, Dublin, Ireland

**Keywords:** Electrospinning, Nano Fabrication, Energy Harvesting, Self-Powered Sensing, Wearable Electronics, Wearable Energy Storage, Wireless Communication, Textile Engineering

## Abstract

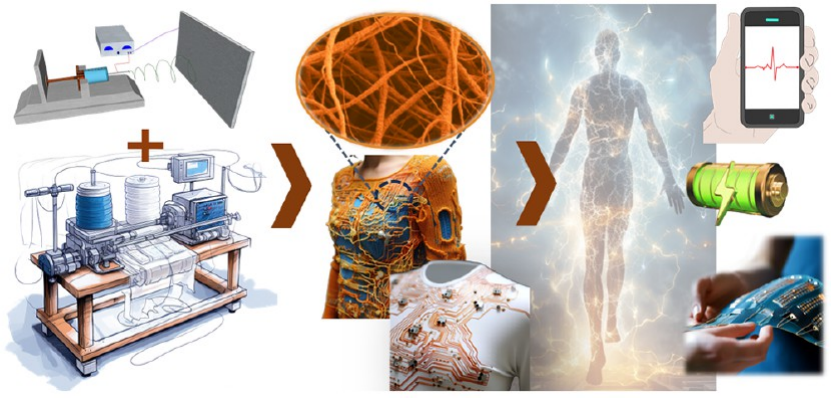

The market for wearable electronic devices is experiencing significant growth and increasing potential for the future. Researchers worldwide are actively working to improve these devices, particularly in developing wearable electronics with balanced functionality and wearability for commercialization. Electrospinning, a technology that creates nano/microfiber-based membranes with high surface area, porosity, and favorable mechanical properties for human *in vitro* and *in vivo* applications using a broad range of materials, is proving to be a promising approach. Wearable electronic devices can use mechanical, thermal, evaporative and solar energy harvesting technologies to generate power for future energy needs, providing more options than traditional sources. This review offers a comprehensive analysis of how electrospinning technology can be used in energy-autonomous wearable wireless sensing systems. It provides an overview of the electrospinning technology, fundamental mechanisms, and applications in energy scavenging, human physiological signal sensing, energy storage, and antenna for data transmission. The review discusses combining wearable electronic technology and textile engineering to create superior wearable devices and increase future collaboration opportunities. Additionally, the challenges related to conducting appropriate testing for market-ready products using these devices are also discussed.

## 1 Introduction

Rapid advances in wearable technology have influenced many industries, including healthcare, sports, safety, environmental monitoring, space exploitation, soft robotics, transportation, and industrial sensing. The uptake of such technology calls for innovative means of powering such devices, leading to the emergence of energy-autonomous wearable wireless sensing systems providing continuous and reliable monitoring capabilities without requiring external power sources or frequent battery replacements.^1−4^ The concept of energy-autonomous wearable wireless sensing systems has evolved significantly over the years, driven by advancements in materials science, electronics, and wireless communication technologies. While early wearable sensors focused mainly on simple sensing functions, such as heart rate, respiration, body movement, and step counting,^4−6^ recent developments have enabled more sophisticated functionalities (e.g., real-time multiple physiological parameter monitoring, motion tracking, machine learning augmented brain–computer interfaces, and environmental sensing). In addition to these more advanced sensing functionalities, the potential to harnessing energy from the environment has been addressed in order to provide longer and more continuous operation while wirelessly transmitting data and information.^7,8^ With the continuous evolution of the Internet of Things, artificial intelligence and 5G/6G technologies, wearable electronics could play a pivotal role with a market value of 150 billion Euros by 2028.^9^ Balilonda et al. reported that the market for wearable sensors is expanding at a compound annual growth rate of 18%, with a projected value of USD 265.4 billion by 2026.^10^ This demonstrates the increasing demand for wearable technologies across multiple industries, which will require advance energy-autonomous systems. The ability to eliminate or reduce the need for battery replacements in wearable devices would greatly enhance their convenience, usability and sustainability, thereby making them more appealing to consumers and industries.

**Figure 1:**
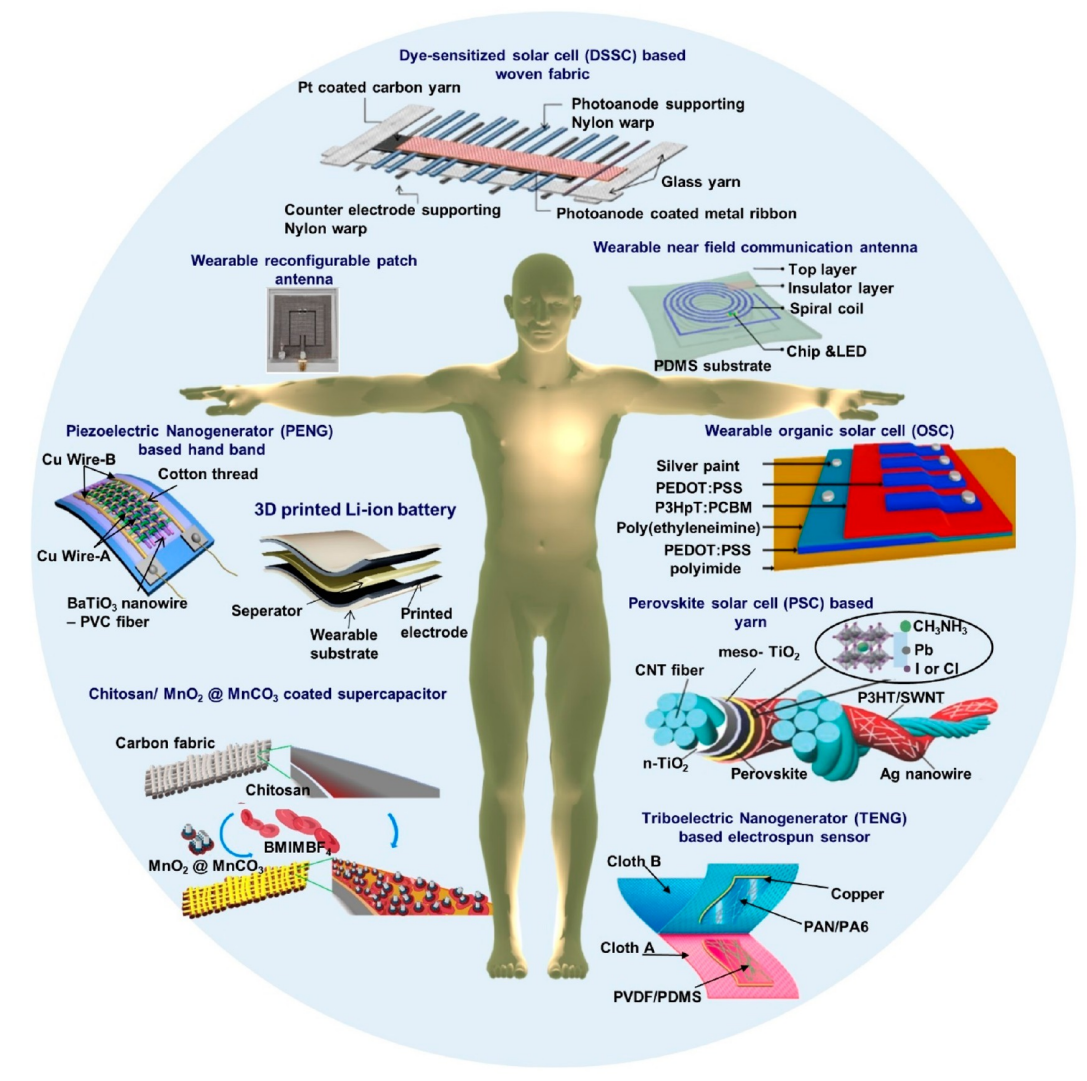
Examples of recent developments in energy autonomous wireless sensing devices including wearable energy harvesting, self-powered sensing, energy storage, and communication devices. PENG, reprinted from ref (37), and OSC, reprinted from ref (38) with permission. Copyright 2015 Elsevier. PSC, reprinted from ref (39) with permission. Copyright 2015 Wiley-VCH Verlag GmbH & Co, TENG, reprinted from ref (40) with permission. Copyright 2017 Elsevier. DSSC, reprinted with permission under a Creative Commons [CC BY] License from ref (41). Copyright 2015 The Authors. Published by Springer Nature. Supercapacitor, reprinted from ref (42) with permission. Copyright 2022 Elsevier. Patch antenna, reprinted with permission from ref (43). Copyright 2018 IEEE. Li-ion battery, adapted with permission from ref (44). Copyright 2020 Elsevier Ltd. Near-field antenna, reprinted with permission under a Creative Commons [CC BY] License from ref (18). Copyright 2020 The Authors. Published by Springer Nature.

Creating energy-autonomous wearable wireless sensing systems involves the integration of several essential building blocks (Figure 1). Such blocks include (i) energy harvesters, (ii) energy storage devices, (iii) sensors, (iv) communication modules, and (v) processing units.^11^ Energy harvesters are responsible for converting ambient energy sources into electrical energy that can be used to power the wearable device. The ambient energy may be harvested from sources including solar (organic solar cell (OSC), perovskite solar cell (PSC), dye-sensitized solar cell (DSSC)),^12^ mechanical (piezoelectric nanogenerator (PENG), triboelectric nanogenerator (TENG)),^13^ or thermal energy.^14^ The energy storage devices, such as batteries, supercapacitors, and hybrid systems, store the harvested energy for use when ambient energy is unavailable.^15^ This ensures the continuous operation of the wearable device. Sensors play a crucial role in collecting data from the surroundings. This data way emanates from the environmental conditions around the wearer or from physiological signals from the wearer’s body. Moreover, these data provide useful information about the wearer’s status for various applications in health, lifestyle, safety, and work and provide additional input methods for human–machine interfacing. In addition, this data from the sensors must be transferred from the wearable device to external devices. Therefore, communication modules are required to facilitate wireless data transmission to external devices such as smartphones or laptops which may be connected to networks for further processing, data storage, and analysis. In addition, several processing units are typically required within the wearable device to perform preliminary data processing before data transmission.^16,17^

The characteristics of the human body, such as its shape, comfort requirements, and safety concerns, presents challenges to the development of wireless sensing systems for wearable applications. To ensure comfort and wearability, wearable devices must be flexible, lightweight, breathable, biocompatible, and capable of conforming to the contours of the human body.^1^ Furthermore, these devices require robust and reliable fabrication techniques for scalable production while maintaining high performance and functionality. This necessitates the exploration of different materials and manufacturing approaches to meet these requirements and enable seamless implementation of the systems on the human body.^16^ Researchers have been experimenting with a range of materials and manufacturing techniques to develop energy-autonomous wearable wireless sensing systems. These include traditional polymers, advanced metallic and functional materials (summarized in section 2.3), and processes like lithography, casting, printing, and chemical and mechanical modifications.^18−21^ While these methods typically deliver satisfactory electrical and sensing performance, there are still challenges to overcome with regard to the wearer’s comfort during everyday body movements. To create truly wearable devices, experts are exploring advanced nanofabrication techniques and textile engineering concepts for enhanced scalability.^22^ Techniques that are compatible with large-scale textile manufacturing processes are essential to bring these concepts beyond the lab and into feasible production lines.

One approach is to use nanofabrication techniques to integrate sensing and electrical properties into fibers and fabrics. Among these methods, electrospinning has emerged as a plausible candidate for human *in vitro* and *in vivo* applications.^23,24^ Various techniques such as printing, sputtering, spin coating, and chemical vapor deposition^25^ are employed to fabricate conductive substrates for wearable electronics. However, the application of mechanical stress such as stretching or bending can lead to the formation of microcracks, which can ultimately lead to a decrease in the conductivity of the electrodes.^26^ Interestingly, the electrospinning technique offers a multitude of benefits over other film processing methods by creating a micro/nano porous fiber structure for the development of energy-autonomous wearable sensing systems. It allows for multiple fiber alignments and customizable porosity targeting flat and asymmetric surfaces^27^ and boasts a high surface-to-volume ratio for sustainable manufacturing.^28^ Its molecular-level alignment reduces the need for postprocessing techniques, such as *in situ* formation of piezoelectric properties in poly(vinylidene fluoride) (PVDF) and its copolymers.^29−31^ Additionally, it enables multicomponent nanofabrication in a single micro/nanoscale step.^32,33^ Compared to other techniques such as photolithography, chemical vapor deposition, and inductive couple plasma etching, electrospinning is more cost-effective and efficient, making it a plausible choice among the scientific community.^34^ Furthermore, electrospun nanofibers can be easily formed into yarn via twisting and braiding techniques and then converted or attached to fabrics through weaving, knitting, or embroidery, making it an ideal method for integrating with the current textile manufacturing processes.^35,36^ All of these factors contribute to the positive impact of electrospinning, making it the preferred choice for a variety of applications. Additional advantages of electrospinning are discussed below.

### 1.1 Fiber-Based Structure

Electrospinning enables the fabrication of ultrafine/intricate three-dimensional fiber networks with diameters ranging from nanometers to micrometers. These fibers can be easily collected as nonwoven mats or aligned into patterns with desirable wearable properties, such as breathability,^45−47^ washability,^48^ biocompatibility,^45^ stretchability, and flexibility.^49^ These fibers can serve as construction blocks for a variety of components, including sensors, electrodes, and energy storage elements.^35^ Especially, due to the ultrafine fabrication nature, electrospinning can produce breathable, washable, transparent, and flexible graphene-based electrodes^50^ which are comparatively cost-effective compared with Ag- or Au-based nanostructured electrodes. The high surface area to volume ratio of electrospun fibers provides enhanced sensitivity for sensing applications and efficient charge storage for energy-related devices.^51^

### 1.2 Controllability of Properties

The process can be easily modified to control the fiber morphology, composition, and alignment. By adjusting the spinning parameters and using different materials, it is possible to create fibers with tailored properties such as high porosity, surface area, tensile strength, and elastic modulus.^12,28,36,52−54^ Furthermore, advances in some electrospinning techniques such as needleless electrospinning, wet electrospinning, and blow electrospinning have provided higher output targeting a shorter manufacturing time.^32,55−59^ This versatility renders electrospinning suitable for a wide range of wearable electronic applications.^12,28,36,52,53^

### 1.3 Integration with Flexible Substrates

Wearable electronics require flexible and conformable substrates to ensure comfort and functionality. Electrospinning can be performed directly onto flexible substrates, including yarns, fabrics, and polymer films, without requiring complex processing steps. The resulting electrospun fibers can conform to the substrate’s surface, allowing for seamless integration with textiles, apparel, and even directly onto the human body. This conformability enhances the comfort and wearability of the electronic devices.^36,60^ Selecting suitable filler materials,^61^ using bicomponent^62^ or multicomponent electrospinning techniques^63^ and introducing further processing methods,^64^ flexibility, elasticity, and other related mechanical properties can be further enhanced.

### 1.4 Multifunctionality

Electrospinning allows for the incorporation of various functional materials into the fibers. By combining different polymers, nanoparticles, or even biological molecules, electrospun fibers can exhibit multiple functionalities such as conductivity, biocompatibility, and biodegradability. This multifunctionality makes electrospinning an attractive technique for fabricating complex, integrated wearable electronic systems.^12,28,36,52,53,65^

To date, there have been some review papers written in the context of electrospinning technology and its implementation. For example, Xue et al. comprehensively reviewed the process of electrospinning nanofibers, methods, and applications.^51^ Recently Zhi et al.,^36^ Babu et al.,^28^ and Joshi et al.^66^ have provided comprehensive reviews on the use of electrospinning in piezoelectric-, triboelectric-, and supercapacitor-based wearable devices, respectively. In contrast to previous research, this paper provides an in-depth review of how electrospinning contributes to the development of different building blocks in wearable, wireless, energy-autonomous devices and considers how to integrate them using traditional textile engineering techniques. Articles included in this review are focused on applications in energy-autonomous wearable wireless sensing systems until mid-2023. In addition to the improvement of energy harvesting, energy storage, sensing, and transmission, equal attention has been given to the mechanical and aesthetic performance improvements using different fabrication techniques. To our knowledge, such a review combining all these perspectives of technology, materials science, textile manufacturing, and user requirements has not been reported previously.

The following review is structured as five comprehensive sections. Section 2 provides a detailed explanation of the history, working mechanism, and optimization parameters of electrospinning technology, as well as an overview of the current state-of-the-art in this field. Section 3 delves into the applications of electrospinning technology in mechanical, solar, thermal, and evaporative energy harvesting, as well as self-powered sensing. In addition, we discuss the development of wearable storage devices in section 3.5, while section 3.6 focuses on optimization strategies for wearable antenna development techniques, without sacrificing performance or wearability. Section 4 provides valuable insight into the scalability of an electrospinning-based wearable energy-autonomous wireless sensing system, using conventional textile engineering concepts. Additionally, section 5 addresses the currently available standard testing procedures for wearable applications, aimed at producing market-ready products. Finally, section 6 briefly discusses the future research avenues, applications, and challenges within the field.

## 2 Electrospinning Technique

### 2.1 Brief History

Electrospinning can be categorized as a form of electrostatic spraying. Electrostatic spraying applies a small charge to an aerosolized droplet before it separates from the nozzle. Electrospinning utilizes this approach to produce continuous fibers through jet formation. The fiber properties are determined by the viscosity and viscoelastic properties of the polymer material.^51^ Although the history of electrospinning dates back to the early 1600s, it was not until 1902 that Mortan and Cooley filed multiple patents for the electrospinning process.^35^ In 1938, the Soviet Union began using the commercially available electrospun product to capture aerosol particles. Between 1964 and 1969, Geoffrey Ingram Taylor conducted a series of experiments to mathematically understand the cone-shape polymer droplet under applied high voltage.^35,67,75^ In 1996, Reneker et al. reported the possibility of producing nanofibers using the electrospinning technique, attracting the interest of scientists worldwide as a promising nanofabrication technique.^68^

### 2.2 Operating Principle

**Figure 2:**
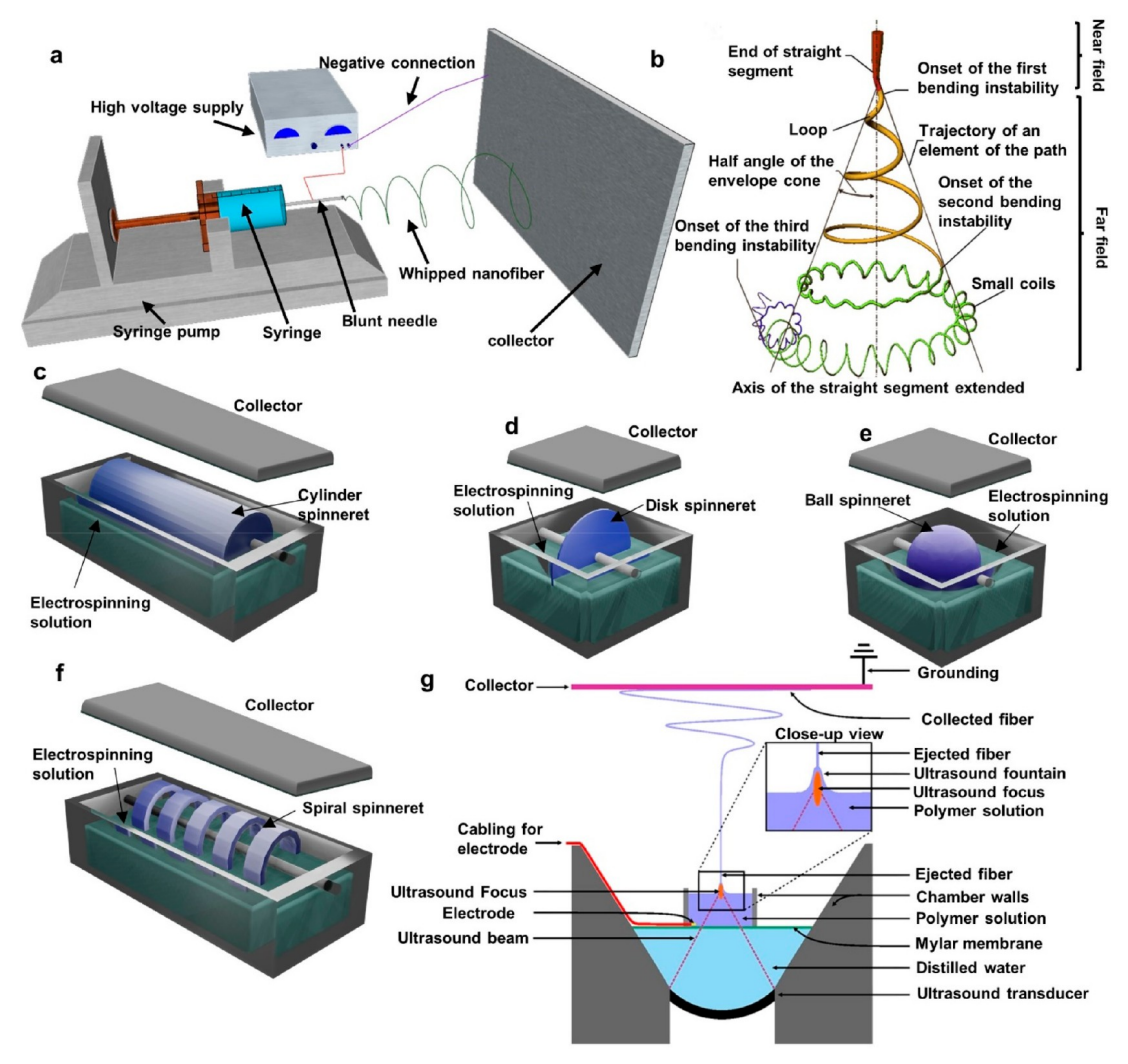
Principle and evolution of electrospinning technique. (a) Basic electrospinning setup and (b) electrospinning whipping action. Reprinted from ref (51 and 69) with permission. Copyright 2006 American Chemical Society. Needleless electrospinning with rotation: (c) cylinder, (d) disk, (e) ball, and (f) spiral techniques. c–f are adapted with permission under a Creative Commons [CC BY] License from ref (70). Copyright 2012 The Authors. Published by Hindawi Publishing Corporation. (g) Ultrasound-enhanced electrospinning technique use with polymer chamber reprinted with permission under a Creative Commons [CC BY] License from ref (71). Copyright 2018 The Authors. Published by Springer Nature.

A basic electrospinning setup consists of three main parts: the jet formation mechanism, the collection mechanism, and the high-voltage source^72^ (Figure 2a). Typically, the jet formation mechanism comprises a polymer-loaded syringe with a blunt needle tip. The majority of materials used in electrospinning are organic polymers. The possibility to dissolve some of these polymers in an appropriate solvent meets the lead requirement for implementing the solvent/solution-based electrospinning process.^51^ Certain polymer materials have high chemical resistance, which has led to the development of a technique called melt electrospinning. This technique involves melting the polymer and using it for melt-blowing and spinning bonding to produce nanofibers. The polymer is compressed through this needle using a controlled mechanism. The high voltage applied between the needle tip and collector causes charges to accumulate around the tip and the polymer droplet. When the electrostatic repulsion force created by accumulated charges exceeds the surface tension defined for a particular polymer type, nanofibers begin to attract toward the collector, followed by solidification and deposition in a randomly oriented way.^72,73^ Initially, the repulsive force converts the polymer droplet into a cone-like shape, commonly referred to as Taylor’s cone. Even though the jet is formulated as a straight line, it undergoes rigorous whipping due to bending instabilities (Figure 2b). Solution parameters, process parameters, and ambient environmental parameters have a significant impact on the performance of the electrospinning process.^74^ The effects of each parameter on nanofibers formation are summarized in Table 1.

**Table 1:** Effect of Solution, Process, and Ambient Environment Parameters for the Electrospun Nanofibers

parameter	effect on the nanofibers
Solution Parameters	
Solvent evaporation rate	Determines the solidification rate. Mainly affected in solution electrospinning. Clogging at the needle might occur when the volatility is very high. Low evaporation causes wet fibers to form at the collector, resulting in solvent patches.^51^
Solvent dielectric constant	Influences the magnitude of electrostatic repulsion at the jet; the higher the dielectric constant, the higher the applied voltage required for stable jetting. A higher dielectric constant reduces the interfiber spacing.^77^
Solubility	High solubility is always favorable for preparing fine fibers.^72^
Polymer type	Determines the selection of molecular weight, viscosity, solution type, and concentration, as well as the process parameters.
Polymer concentration	Determines the consistency of the formulated fiber network depending on the type of material and its molecular weight. If the concentration is too low, the fibers tend to either discontinue or merge.
	For instance, poly vinyl alcohol (PVA) 5 wt % resulted in beaded fibers, 15 wt % resulted in uniform fiber, 25 wt % resulted in coarse nonuniform fiber.^78^
	Polyamide (PA)6 6 wt % resulted in droplets, 15 wt % resulted in merged fibers, 25 wt % resulted in smooth fibers.^79^
	If the concentration is too low, electrospraying is prominent with discontinuous, merged or bearded fibers. In needleless electrospinning techniques, a very high concentration completely halts the electrospinning process.^60^
	In cellulose acetate (CA) nanofibers, the tensile strength, break strain, and initial modulus increase with increasing concentration.^80^
Viscosity	Depending on material type, solvent type and concentration decreasing viscosity and surface tension results in thinner fibers.
	High viscosity complicates the ejection process. A minimum viscosity is always necessary for chain entanglement.^81^
Molecular weight	Affects the viscosity of the polymer solution.^82^ Low molecular weight and limited chain entanglements produce nanofibers with beaded structure; the fiber diameter increases with molecular weight.^78^
Conductivity	If the solution is completely insulating, electrospinning is not possible. High conductivity reduces the diameter of fibers.^72^ The Taylor cone will not develop if the conductivity is too high.^51^
Process Parameters	
Applied voltage	Sufficient voltage is required to compensate for the repulsive force caused by surface tension. Average fiber diameter decreases as the voltage is increased further. Additionally, the change of α to β phase while high voltage is rapid, which favors energy harvesting applications. Further increasing voltage results in beads and fiber breaks due to increased drawing stress.^82^
	In needleless electrospinning, high voltage reduces fiber diameter and increases fiber production rate.^60^
Needle gauge	Affects the diameter of the fibers. Additionally, different gauges can result in multiple jets.^28^
Spinneret types	Conventional method—blunt needle.
	Advanced developments—needleless; cylinder, ball, porous tube, disk, coil, cone, stepped pyramid, wire frame, cleft, bead chain, bowl, slit^23^ (see section 2.4).
Flow rate	A high flow rate results in higher fiber production and coarser fibers; however, an excessively high flow rate results in droplets without the formation of fibers. Flow rates lower than the critical value (value which produces the jet with usual Taylor cone), on the other hand, cause congestion at the needle tip with an unstable jet, branching splitting, and flattened fibers.^60^
Collector design	Variations in collector design and its effects.
	Metal plate collector: a simple architecture to achieve uniform morphology.^72^
	Double plate collector: better alignment than metal plate collector.
	Circular electrode collector: high productivity with easy separation of membranes.
	Rotating cylinder: fiber alignment is high with reduced diameter and high physical properties. Further, increase the β phase of materials such as PVDF which is favorable in energy harvesting and self-powered sensing applications.
	Rotating disk collector: highly aligned nanofibers with enhanced β phase.^82^
	There are some other techniques such as liquid bath, guide wire, rotating wire drum and conveyor,^23,83^ which can be used to produce nanofibers in continuous uniform operation.
Tip to collector distance (TCD)	Mainly affects the evaporation process; the higher the distance, the higher the evaporation and stretching. However, over the optimum value, electric field intensity decreases. Additionally, a higher value will prevent the deposition of fibers onto the collector. A lower distance will result in a denser structure.^82^
	In needleless electrospinning, short distances provide interconnected nanofibers with outstanding mechanical properties.^60^
Ambient Environment Parameters	
Relative humidity	Low humidity is preferable for uniform morphology.^72^
Temperature	Increasing the temperature increases the rate of solvent volatilization and decreases viscosity and surface tension, resulting in the formation of small diameter fibers.^82^

In 1969, Taylor derived an equation to approximately determine the critical voltage (*V*_k_, kV) required to overcome the surface tension in a given material.^75^
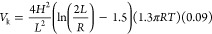
1

In eq 1, *H* (cm) is the distance between the needle tip (or spinneret) and the collector, *L* (cm) is the length of the needle (or spinneret), *R* (cm) is the outer radius of the needle, and *T* (dyn/cm) is the surface tension of the polymer material. Based on this equation, there is a proportional relationship between the surface tension and the critical voltage at the jet formation. The value of 1.3 was determined using the assumption that the cone has a semivertical angle with a value of 49.30 (2 cos (49.3) = 1.3). The factor 0.09 was inserted to get the outcome in kV. Viscoelastic properties of the polymer material should ensure that the continuous fiber surpasses the Rayleigh limit after the near-field region.^51,76^ It is worth noting that one of the important phenomena in nanofiber formation is radial charge repulsion, which allows for whipping action.

### 2.3 Materials

In the literature dimethylformamide (DMF),^80,84^ dimethylacetamide,^80^ certain alcohols,^51^ formic acid, dichloromethane,^85^ tetrahydrofuran,^80^ chloroform,^86^ acetone,^87^ hexafluoroisopropanol,^73^ dimethyl sulfoxide,^88^ and methanesulfonic acid^89^ are widely used as solvents for electrospinning. Despite the fact that water is not a favorable solvent for electrospinning due to its high dielectric constant, PVA electrospinning is generally carried out using DI water.^51,90^ Furthermore, a wider variety of biodegradable and biocompatible polymers can be electrospun, targeting wearable electronic applications. By utilizing electrospinning, it is possible to enhance the mechanical properties of biodegradable materials, including increased flexibility, stretchability, and breathability when compared to film-based substrates.^91^Table 2 summarizes the use of different materials for electrospinning, mainly in wearable contexts, which is the focus of this review paper. Different electrospinning techniques for improving the performance related with wearable applications using these materials have been detailed in the sections below.

**Table 2:** Prominent Electrospinning Materials in the Building Blocks of Energy Autonomous Wireless Sensing Systems

application	mechanism	electrospinnable layer	electrospinning materials
Energy harvester	TENG, TENG self-powered sensors	Triboelectric layer	PVDF,^82^ polyimide (PI),^92^ polyvinylidene fluoride-trifluoroethylene (PVDF-TrFE) and poly(vinylidene fluoride-hexafluoropropylene) (PVDF-HFP),^93^ poly lactic acid (PLA), CA, PA6, PA66, MXene^28^
		Electrode layer	Poly(3,4-(ethylenedioxy)thiophene) (PEDOT), carbon nanotubes (CNT)^1,28^
	PENG, PENG self-powered sensors	Piezoelectric layer	PVDF, PVDF-TRFE, PVDF-HFP, polyacrylonitrile (PAN), cellulose, PLA^94^
		Electrode layer	PEDOT, CNT, polyaniline (PANI)^36^
	DSSC	Electrode layer	Pt, ZnO, ZnO-TiO_2_
		Supporting materials	PLA, PVDF and PVA, PVP, CA
			Cu(In_1–*x*_Ga_*x*_)Se_2_, Cu_2_ZnSnS_4_, and Cu_2_ZnSnSSe_4_^95^
	OSC	Electrode layer	PAN, PANI^96^
		Functional/supporting material	Poly(methyl methacrylate),^97^ polycaprolactone (PCL), poly(3-hexylthiophene):phenyl-C61-butyric acid methyl ester (P3HT:PCBM)^98^
	PSC	Supporting material for perovskite layer	Polyvinylpyrrolidone (PVP),^99^ PI, polyurethane (PU)^100^
Storage	Supercapacitors	Supporting material for electrolyte layer	PLA, PVDF, PVA^101^
		Electrode layer	PEDOT, PANI, polypyrrole (PP), carbon nanofibers (CNF)^66^
	Li-ion batteries	Electrode layer	PEDOT:PSS, polydopamine, PP, carbon nanofiller reinforced cellulose^66^
		Supporting material	PLA, PVDF, and PVA
Communication	Antenna	Radiating layer	Ag nanoparticles, ethylene glycol^18,53^
		Support of radiating layer	PVA

### 2.4 Advanced Electrospinning Techniques

Centrifugal spinning has been employed for over half a century to fabricate glass nanofibers by regulating parameters such as the radius of the orifice, angular velocity of the spinneret, distance from the center of the orifice to the collector, polymer-related parameters, and environmental conditions. Centrifugal electrospinning is an advanced architecture that applies an electrostatic field to the traditional centrifugal spinning technique. Experimental results demonstrate that the production rate of centrifugal electrospinning is 12 times that of conventional electrospinning systems with low polymer concentration.^32^ In addition, centrifugal electrospinning promotes an exceptional improvement in mechanical performance and, in conjunction with melt electrospinning, can produce ultrafine fiber for high-rate supercapacitors.^32,102^

Earlier advances in electrospinning technology indicate that multinozzle electrospinning and multicomponent electrospinnable nozzles result in increased throughput and enable functional material developments.^32,33^ Using a multinozzle electrospinning technique and moving collector apparatus, such as rotating drum, mandrel, or belt, it is possible to develop highly oriented nanofiber architectures with high mechanical qualities for wearable applications. In addition, multicomponent techniques can produce bilateral conductive and nonconductive surface samples necessary for energy harvesting, storage, and communication devices.^32,33^

Even though traditional needle-based electrospinning is a simple and versatile nanofabrication technique, low production yield, needle clogging, and limited capacity have limited its practical applications.^24^ Moreover, the rapidly growing wearable electronics market necessitates a higher production rate, mandating alternative electrospinning techniques. Needleless electrospinning is a popular method, which uses a widely open liquid surface with a specially developed spinneret that is partially submerged in the electrospinning solution. As spinnerets for needleless electrospinning, cylinders (Figure 2c), disks (Figure 2d), balls (Figure 2e), springs (Figure 2f), coils, wires, rods, and spirals are increasingly popular. All these spinnerets can create a thin polymer layer on their surface as a result of rotation-induced agitation. Based on the intensity of the electric field, a conical spike is formed toward the collector, resulting in multiple polymer jets. Some experiments provide evidence that ball and disk spinnerets have good control over fiber diameter and productivity, whereas changing the electric field in ring and coil spinnerets leads to thinner fibers with a large surface area.^32^

In 2016, Laidmäe et al. filed a patent for an ultrasound-enhanced electrospinning technique (Figure 2g).^71^ This method used a focused and highly intense ultrasound to create an ultrasonic fountain when a precursor solution was placed on a Mylar (electrically insulating and acoustically conducting) membrane. Controlling ultrasound parameters and maintaining sufficient electric field toward the collector can control the gradients of mechanical properties, giving greater prospects for future wearable electronic devices.^71,103^

Typically, electrospinning falls within a flow rate range of 4–10 μL/min, which presents challenges for scaling up production. However, blow spinning is a promising and relatively unexplored method that can inject fibers at a much higher rate of 200 μL/min. This technique leverages gas pressure to propel the polymer onto the surface, similar to melt spinning, and combines it with the polymer dissolution process used in solution electrospinning.^55,56^ Blow spinning has shown promise in producing microscale fibers for wearable electronic applications.^104^

Mostly, electrospun membranes feature tightly packed small pore sizes formed from small-diameter nanofibers. However, by transitioning from traditional solid collectors to grounded liquid baths (using a nonsolvent of electrospun polymer), it becomes possible to achieve liquid-phase collection with specific functionalities, such as high porosity with 3D morphology.^105,106^ This method, known as wet electrospinning, is an area that has been relatively unexplored in wearable electronic applications. Nonetheless, evidence suggests that the 3D morphology of this technique facilitates rapid access to electrolytes in energy storage devices, resulting in faster charge–discharge times and higher storage capacity.^57−59^

## 3 Electrospinning Implementation for Energy-Autonomous Wearable Wireless Sensing System Development

This section discusses the application of electrospinning techniques to manufacture textile compatible materials for energy harvesting and self-powered sensing functionalities. Sections 3.1 and 3.2 explore the use of electrospinning for mechanical energy harvesting and self-powered sensing, respectively, using triboelectric and piezoelectric techniques.

### 3.1 Electrospinning-Enabled Wearable Mechanical Energy Harvesters

**Figure 3:**
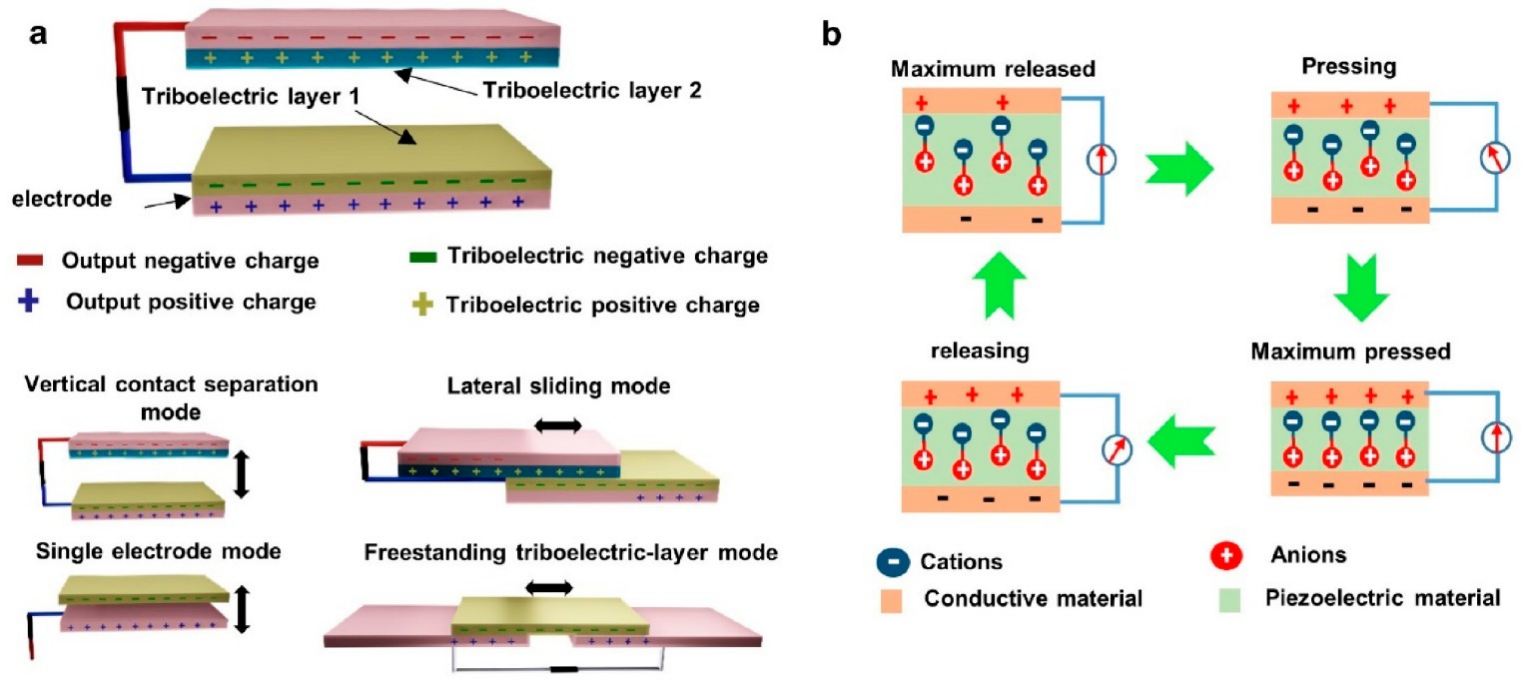
Principle and working mechanism of mechanical energy harvesting techniques. (a) Schematic of TENG and main working modes and (b) working mechanism of PENG. Adapted from ref (118) with permission. Copyright 2021 American Chemical Society.

Movement from the human body is a pertinent energy source for powering wearable sensors. For instance, the movement of ankle, arm, knee, shoulder, elbow, and fingers can produce 66.8, 60, 36.4, 2.2, 2.1, and 6.9 W to 19 mW of energy, respectively.^107^ The two most widely used methods of wearable energy harvesting based on piezoelectric and triboelectric principles/TENG and PENG devices, shown in Figure 3a,b, respectively, are pioneering mechanical energy harvesting and self-powered sensing techniques which can convert irregular and low-frequency human movements. The conversion of mechanical motion into electrical power/signals can be described by Maxwell’s equations of displacement current.^108,109^ PENG is a concept that was developed in 2006 by Wang’s research group, which works on the stress state and electrical polarization of a specific piezoelectric material.^110−112^ Several years later, Fan, Tian, and Wang developed the TENG in 2012 based on contact electrification and electrostatic induction.^113^ A recent survey shows that over 6000 scientists worldwide are working on TENG research, making it a promising method for wearable electronics power/signals.^108^ Furthermore, based on material selection, architecture, fabrication technique, and power management methods, the power conversion efficiency and peak power outputs of piezoelectric and triboelectric devices can be adjusted.^114,115^Supplementary Note 1 provides some examples related to use of electrospinning in PENG and TENG applications along with functional and wearable characteristics. Additionally, utilizing the electrospinning technique to create hybrid PENG and TENG devices offers advantages due to the natural polarization of certain materials. This results in increased power generation and sensitivity compared to other manufacturing methods.^116,117^

#### 3.1.1 Triboelectric-Based Energy Harvesting

Triboelectric nanogenerators function through triboelectrification and electrostatic induction, resulting in a relative movement of two charged surfaces. Even though the exact process of triboelectrification is still unclear,^108^ evidence claims that it occurs primarily through electrons,^119^ charged materials,^120^ ions, or a combination of all factors.^121^ In addition, Ko et al. examined the electron transfer mechanism of the triboelectrification process and concluded that there is a positive relationship with the potential interface barrier, and stuck charges are the foundation for triboelectric charge separation.^122^ A triboelectric series has been developed based on an empirical classification to identify the positively and negatively charged materials:^123^ e.g., materials such as wood and nylon are at one end of the scale with a tendency to be positively charged while PDMS, PVDF, and silicone rubber have a tendency to become negative. There are a number of publications quantifying the triboelectric series,^123^ and in one of our previous publications by Gunawardhana et al., we present an adjusted triboelectric series related to wearable materials.^1^ A TENG device typically uses two materials that are well separated from each other within the triboelectric series, targeting higher charge separation. There are different setups for arranging the materials withing the TENG devices to ensure the interaction between these materials to generate energy, including contact separation, lateral sliding mode, freestanding electrode mode ,and single-electrode mode. The TENG contact separation mode (Figure 3a) is the most common architecture. Using this traditional architecture, the TENG device has two distinct nonconductive (or one conductive and other nonconductive) materials attached to electrodes which are connected to an external load (Figure 3a). The triboelectric materials undergo close contact and separation movements, resulting in charge separation due to the triboelectric effect. Repetition of relative moment of the materials can induce charge to the attached electrodes, creating electron flow from one electrode to the other through the external load. When the TENG’s triboelectric materials are in contact with each other, the electron flow goes in one direction; then when the triboelectric materials separate the electrons flow in the opposite direction, resulting in an alternating current flow. Selection and fabrication of suitable materials further apart in the triboelectric series, as well as the improvement of the electrostatic induction process, are the pioneering research topics related to this technique.^1^

In an effort to understand the generation of charge within nanogenerators, in 2017 Wang proposed that the current output can be explained using Maxwell’s displacement current theory.^124^
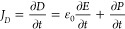
2

In eq 2, *J*_*D*_ is the displacement current density of the displacement field *D* for time *t*, *E* is the electric field, ε_0_ is the permittivity of the dielectric material, and *P* is the polarization field. In this equation, while the term  provides vital information regarding electromagnetic waves in wireless communication,  is directly related to the output current of nanogenerator devices.^108,124^ In addition, the PENG and TENG devices are referred to as capacitive induction devices. A parallel plate capacitor model was developed for contact separation mode TENG architecture to explain the relationship among voltage (*V*), charge (*Q*), and layer separation of nanogenerator (*x*), which is known as the *V*–*Q*–*x* relationship.^124,125^
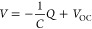
3

In eq 3, *C* indicates overall capacitance while *V*_OC_ indicates the open circuit voltage of nanogenerator devices. Dharmasena et al. developed a distance-dependence electric field (DDEF) model to overcome the limitations of the parallel plate model, such as the complexity of the polarization of dielectric layers, electric field behavior inside the parallel plates, and induction behavior of the output charges in electrodes.^107,126^ This model was developed considering the finite dimension of the charged surfaces and the perpendicular distance (*y*) from the charged surface. According to the DDEF method, the overall electric field (*E*_*Z*_) generated from such a charged surface with a surface charge density of σ and permittivity of ε along with the dimensions of width (*W*) and Length (*L*) can be defined as
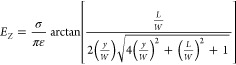
4

The DDEF model can be used not only for charged surfaces but also for analysis of the output behavior of the electrodes. Concurrently, DDEF can predict the power output behavior of the TENG devices. Furthermore, Dharmasena et al. examined the effect of the power output with material parameters, such as triboelectric charge density and dielectric constant, and structural parameters, such as layer thickness and surface area. Theoretical results were conclusive that triboelectric charging has a quadratic relationship with the power output, thus determining charge density as a critical factor in TENG power optimization.^107^ This implies that an increase in surface area and a reduction of the thickness of the TENG layer are favorable for higher power generation in TENG devices. Also, increasing the surface area reduces the internal impedance, and maintaining a sufficient thickness is crucial to stabilize the accumulation of triboelectric charges. Electrospinning can be used in TENG devices to increase the surface contact area, thus increasing charge density and resulting in high power output. In addition, due to the nature of nanofabrication the thickness of the layers produced by electrospinning can be controlled, which is favorable for high power generation.

**Figure 4:**
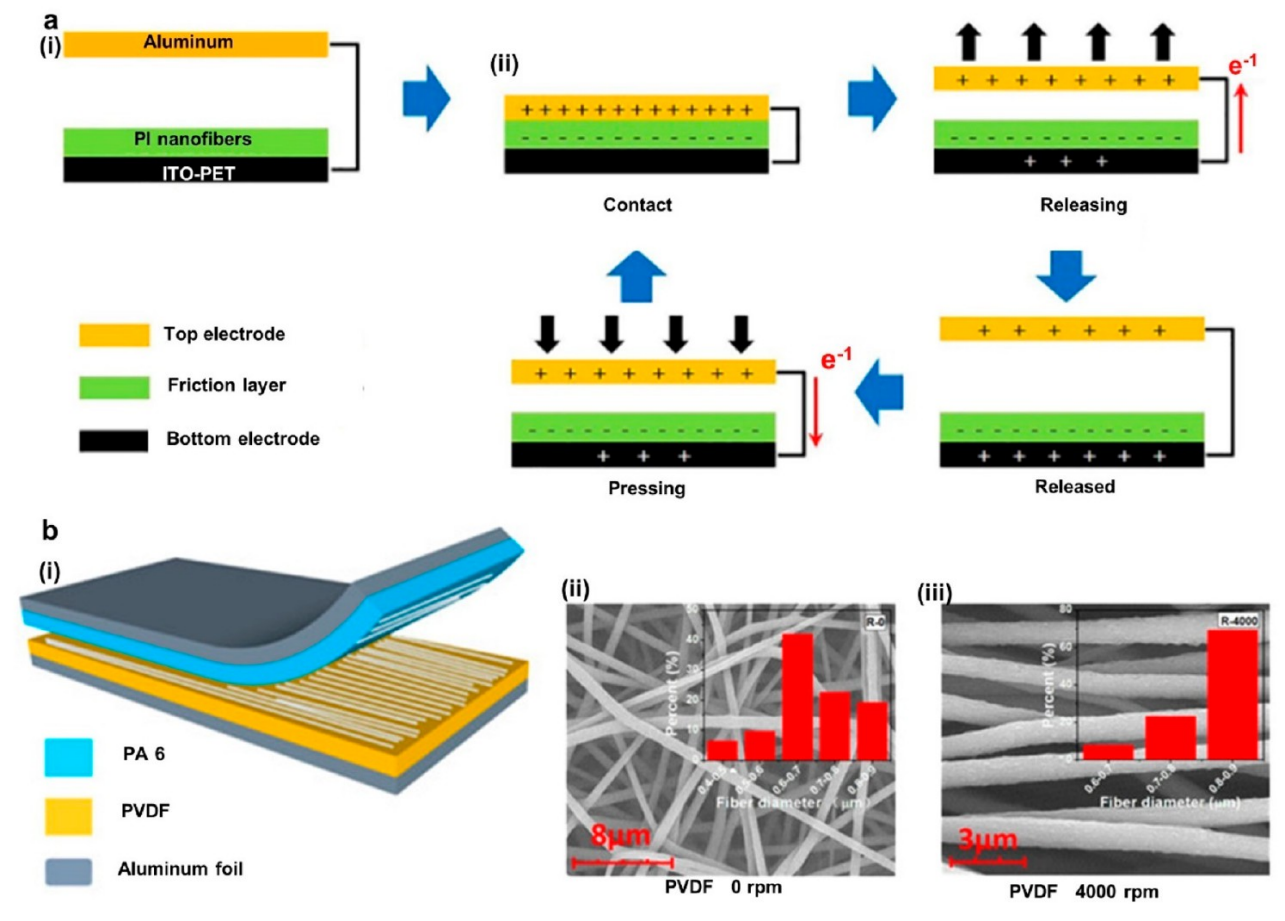
Using electrospinning single and dual triboelectric layer modification for energy harvesting applications. (a) Schematic of PI nanofiber and aluminum-based TENG. Reprinted with permission under a Creative Commons [CC BY] License from ref (92). Copyright 2020 The Authors. Published by Springer Nature. (b) Ordered electrospun sample schematic and SEM images of 0 and 4000 rpm rotary collector based nanofibers, (i)–(iii), respectively. Reprinted from ref (127) with permission. Copyright 2020 American Chemical Society.

Electrospinning can manufacture single triboelectric surfaces or composite-based device architectures to create TENG devices.^28^ Kim et al. have investigated the possibility of developing TENG devices using PI nanofibers in a one-step electrospinning process (Figure 4a). In this experiment, the PI layer was used as the tribonegative layer, while aluminum was used as the tribopositive layer. Initially, this setup demonstrated an open circuit voltage (*V*_OC_) of 66.1 V and short circuit current (*I*_SC_) of 1.68 μA while using commercial PI films. Subsequently, the commercial PI film was replaced with screen-printed PI film which resulted in a reduction of *V*_OC_ to 45.6 V and *I*_SC_ to 1.61 μA. Conversely, electrospun PI nanofiber film demonstrated a significantly higher *V*_OC_ of 366 V and *I*_SC_ of 6.52 μA, thereby increasing the performance of the TENG device. SEM images reveal that the surface of commercially produced PI film and screen printing is flat and has a lower surface area compared to electrospun samples. It is worth noting that there were no noticeable differences in the static electricity of all three samples; therefore, it is the increase in surface area that is attributed to being the main factor in creating higher surface charges and greater electrostatic induction which ultimately leads to higher output power.^92^

The choice of materials is an important factor in the design of TENG devices. Ferroelectric polymers, namely PVDF, PVDF-TrFE, and PVDF-HFP, are widely used in electrospinning-based TENG devices given their high fluorine content which contributes to high electron affinity resulting in tribonegative surfaces.^73^ Lee et al. have published a comprehensive review on the progress of PVDF as a functional material in TENG energy harvesting and self-powered sensing.^93^ To summarize their findings, besides having a high electron affinity, utilizing various electrospinning arrangements can enhance both the physical and electrical qualities of the PVDF fibers produced. Wang et al. have developed TENG using PVDF (tribonegative) and PA6 (tribopositive) with balanced physical and electrical performance using a parallel nanofiber arrangement acquired through electrospinning (Figure 4b). During electrospinning, PVDF was collected through a rotary collector targeting parallel arrangement by changing the rotation speed. The high speed of the drum reduced the fiber diameter and increased the tensile strength in a longitudinal direction. In addition, under 2 Hz impact frequency with a separation of 4 mm, the resulting TENG demonstrated *V*_OC_ of 164 V, *I*_SC_ of 392 nA, and power density of 129.46 mW m^–2^, which was significantly stable for 100000 cycles.^127^ Furthermore, Song et al. observed that increasing the arrangement of electrospun nanofibers in a parallel way can improve the forward polarized dipoles in PVDF, thus increasing *V*_OC_, *I*_SC_, and power density 0.5, 2.6, and 2.2 times, respectively. The occurrence of this phenomenon is highly likely when the charge produced by the piezoelectric feature of PVDF nanofibers aligns with the charge generated by friction. As a result, the surface polarization is significantly increased.^128^

**Figure 5:**
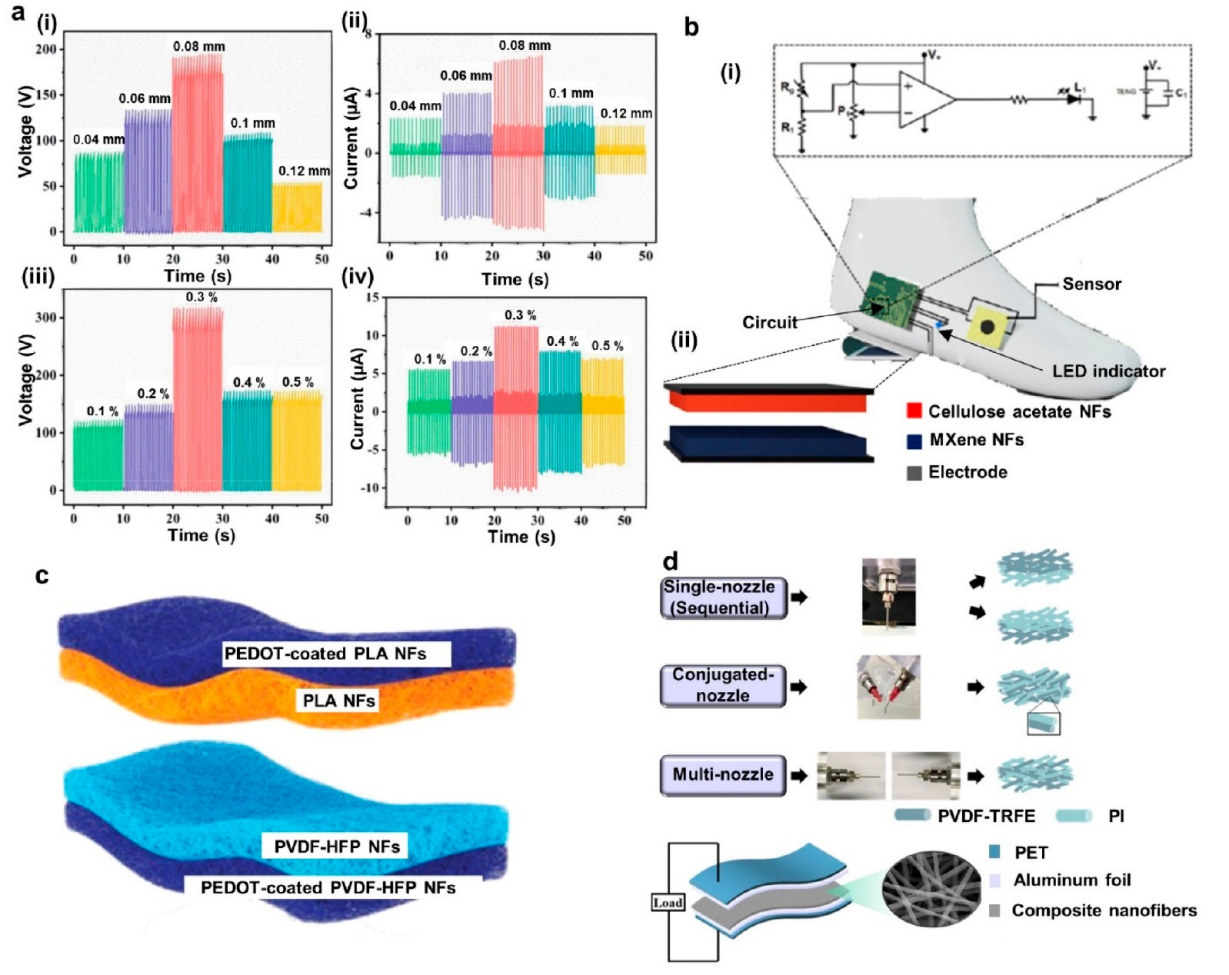
Further improvements and development of composite structures for TENG applications using the electrospinning process. (a) Effect of electrospun PVDF layer thickness ((i) voltage, (ii) current behavior) and MoS_2_/CNT ((iii) voltage, (iv) current) concentration over output performance. Reprinted from ref (130) with permission. Copyright 2022 American Chemical Society. (b) Schematic of electrospun TENG based self-powered NH_3_ monitoring sensor. Reprinted from ref (132) with permission. Copyright 2022 American Chemical Society. (c) Schematic using PLA- and PVDF-based composite TENG device. Reprinted from ref (133) with permission. Copyright 2019 Wiley-VCH Verlag GmbH & Co. KGaA, Weinheim. (d) Schematic of single-nozzle, conjugate-nozzle, and multinozzle needle-based electrospinning systems. Reprinted from ref (134) with permission. Copyright 2021 American Chemical Society.

In addition to pristine material electrospinning, functional filler materials^129^ have been used along with electrospinning precursors to improve the performance of TENG devices. For example, Sun et al. have published a flexible TENG architecture with MoS_2_/CNT (MC)-loaded 12% PVDF electrospun nanofibers (tribonegative) and nylon fabric as a positive layer (Figure 5a). Kelvin probe force microscope results compared with PVDF nanofibers and MC-loaded PVDF nanofibers show that MC can improve the surface potential of PVDF nanofibers, enhancing electrical performances. The 0.3% MC loaded PVDF has resulted in *V*_OC_ of 300 V and *I*_SC_ of 11.5 μA under 50 N contact and separation force with 1.5 Hz frequency. Furthermore, 134 mW m^–2^ power density was achieved through a load resistor of a 100 MΩ resistor and could charge a 10 μF capacitor in 44 s. Contrary to these findings, a further increase of MC content creates a conductive network creating leakage current, thus neutralizing some charges in the surface. Moreover, proving that there needs to be sufficient thickness for charge accumulation and transfer, performance increased when the thickness increased from 0.04 to 0.08 mm (Figure 5a (i, ii)). In contrast, further increases beyond this thickness reduced the performance as given in the DDEF model which we discussed previously. However, the device output was stable over 3000 cycles, and after 6 months of exposure to normal indoor humidity and temperature, 30% performance was retained.^130^

Aside from MC, MXene materials can be used as a filler material, providing excellent electromagnetic interference shielding, high electrochemical activity, and excellent volumetric capacitance properties favorable for TENG applications.^131^ When MXene is used as thin films, there are some challenges to overcome, including low flexibility, a highly brittle nature, and surface roughness. However, electrospinning fabrication can solve these problems by maintaining nanoscale surface roughness, enhancing frictional contact, and increasing surface-to-volume ratio, thus improving flexibility. Sardana et al. developed a wearable real-time gas monitoring system using MXene nanofibers (30% MXene/PVA negative) and 19% CA solution (positive) (Figure 5b). The device (with dimension 3 × 3 cm^2^) could generate a maximum voltage of 140 V, a current of 92 μA, and a power of ∼1.361 W m^–2^ through a 2 MΩ load resistor. The high tribonegativity and conductive nature of MXene have significantly reduced the internal impedance of the triboelectric layers by creating higher outputs. Targeting real-world applications, along with a suitable power management system attached to a shoe insole, the device successfully powered a MXene/TiO_2_/CNFs heterojunction-based sensor for NH_3_ detection^132^ (Figure 5b (ii)).

Electrospinning can be used to develop highly conductive, porous, and flexible electrodes suitable for wearable TENG devices. For instance, Qin et al. demonstrated a composite TENG using a Janus structure with PLA and PVDF NHF nanofiber as triboelectric surfaces and PEDOT to achieve conductivity with the respective layers (Figure 5c (i)). The conductivities of PEDOT–PLA nanofibers and PEDOT–PVDF NHF were recorded as ∼2.63 and ∼66.67 mS cm^–1^, respectively. The device was ultralightweight (2 × 2 cm^2^ sample with 25.8 mg) and demonstrated *V*_OC_ of 140 V, *I*_SC_ of 3.8 μA, a charge of 48 nC, and a peak power of 0.75 mW through 150 MΩ under periodic contact and separation movement. In addition, the device could be used to light up 50 commercial LED lights and identify the throat swallowing and gripping action of the human wrist.^133^ Furthermore, Janus-structured electrospinning creates a breathable, lightweight, and flexible structure that is comfortable to wear. It also improves adhesion between conductive and triboelectric layers while reducing delamination.

Another factor to optimize power output from a TENG substrate is to alter the electrospinning fabrication process parameters. Power output is one of the most important factors that will drive commercialization of this technology. The arrangement of the electrospinning system has a significant impact on the power output of TENG devices. Kim et al. observed the effect of power output based on single nozzle, conjugated nozzle, and multinozzle electrospinning systems (Figure 5d). A PI/PVDF-TRFE composite nanofiber membrane was developed during the experiment using all three techniques. Previous experiments provided evidence that PI has the ability to retain more induced charges and higher electrical properties. Evidence from energy dispersive spectroscopy and SEM shows that using a single nozzle produces a layer-by-layer structure for PI and PVDF-TRFE separately. On the other hand, employing conjugate and multiple nozzle techniques results in a mixture of PI/PVDF-TRFE nanofibers throughout the material, enabling both components to be present and contribute. Based on the results, there was an increase in the power output when using conjugate or multiple nozzles rather than single nozzles, concluding that conjugate or multiple nozzles are a better alternative in the preparation of composite nanofibers. In addition, using a rotary collector instead of a planar collector during multinozzle electrospinning can further enhance the power output of composite-material-based TENG devices.^134^

**Figure 6:**
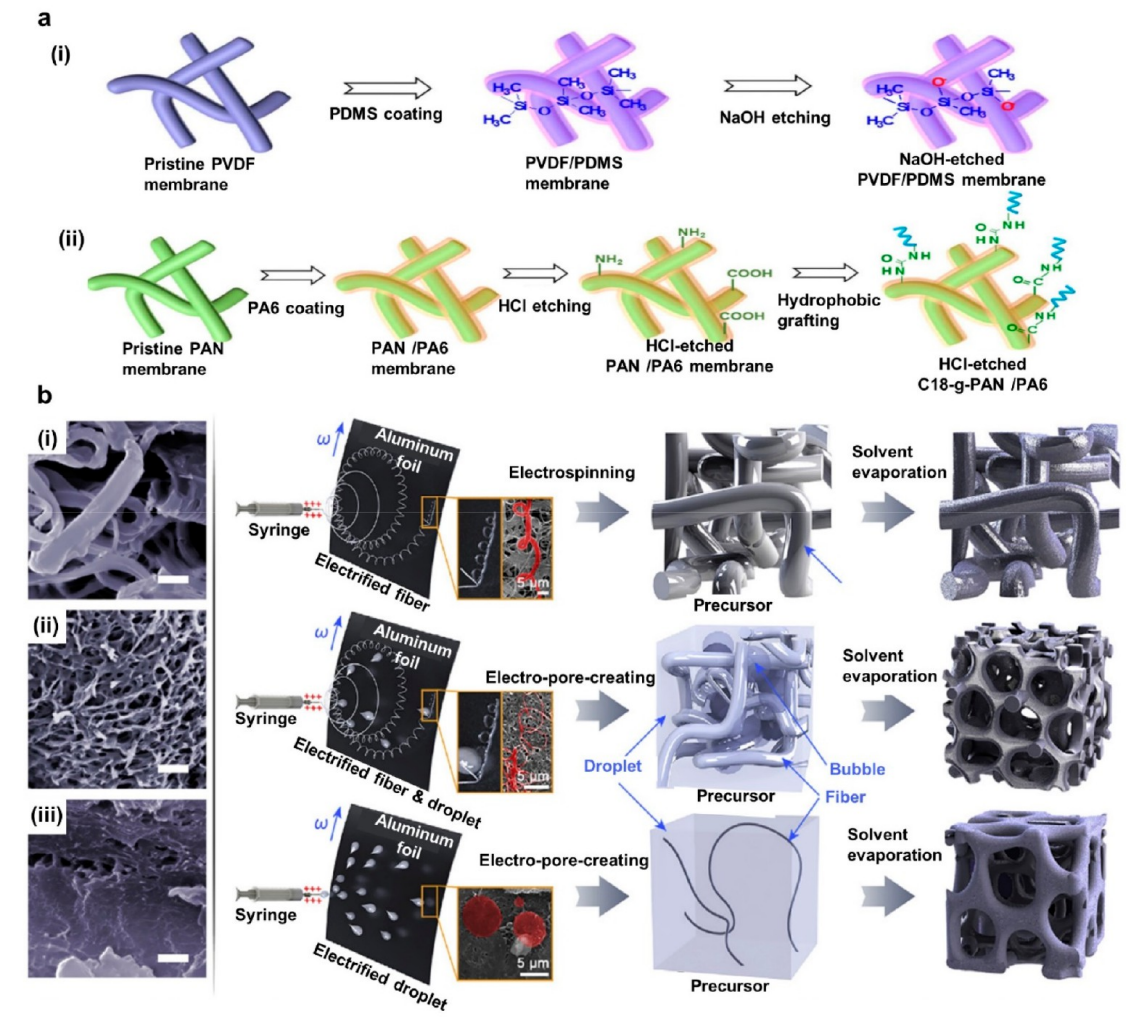
Improving the TENG device’s mechanical, aesthetic, and electrical properties using chemical and mechanical modifications. (a) Schematic of TENG made with an NaOH-etched PVDF/PDMS membrane (i) and HCl-etched PAN/PA6 membrane (ii). Reprinted from ref (40) with permission. Copyright 2017 Elsevier Ltd. (b) Surface modification changing the volatile content of the solvent in electrospinning solution: SEM and preparation of entangled network structure (i), nanoporous cancellous-bone-like structure (ii), and collapsed nanopore structure (iii). Reprinted from ref (30) with permission. Copyright 2019 Elsevier Ltd.

Improving the TENG device’s mechanical, aesthetic, and electrical properties using chemical modification and mechanical modifications is a highly investigated area.^22^ Interestingly, Li et al. observed that the electrospun nanofiber-based TENG device’s mechanical properties, triboelectric polarity, and hydrophobicity could be altered using material design approaches: for instance, coating and etching. When using an NaOH-etched polydimethylsiloxane (PDMS)-coated PVDF membrane (Figure 6a (i)) as the tribonegative material and an HCl-etched PAN/PA6 (C-18 g)-based membrane (Figure 6a (ii)) as the tribopositive material, the device resulted in a *V*_OC_ of 540 V and *I*_SC_ of 110 μA. In contrast, pristine materials show a lower output. In surface-modified samples, *V*_OC_ of 340 V and *I*_SC_ of 60 μA were observed under 90% humidity, while pristine samples demonstrated drastically decayed power outputs. The results of this experiment show the improvement of mechanical properties due to the chemical modification of the electrospun membranes.^40^

As previously discussed, increased contact area resulting in greater surface charge improves the charge generation process. Research has shown that adding a porous structure to the triboelectric frictional layer can increase the effective contact area, which is beneficial for trapping more charge. To optimize the power output of such devices, it is advisible to use a relatively thin layer with moderate porosity (need to determine through experiments), a closed-pore structure with small pore size, and a high dielectric constant.^135^ Zhang et al. investigated the improvement of electrical performance, output stability, and use of comfort by self-assembly electropore creation (Figure 6b). During the experiment, electrospinning solutions (14% PVDF) were prepared, altering the solvent mixture composition with less volatile (dimethyl sulfoxide) and highly volatile (acetone) components. As shown in Figure 6b (i)–(iii), increasing the less volatile content in the precursor has increased the pore size after solvent evaporation of the electrospun sample. Electrospun samples developed with 80% less volatile content in the final solution, contact and separated with a natural rubber mat (area of 20.25 cm^2^) could generate *V*_OC_ of 1403 V and 10.6 W m^–2^ power under 5.5 N force. Under 85% humidity, the device still generates 22% power, and it could accelerate the evaporation of sweating by transferring to the bottom region through the pore structure,^30^ which is an important factor to consider for wearer comfort of such devise in real-world use.

#### 3.1.2 Piezoelectric-Based Energy Harvesting

The piezoelectric property is a natural phenomenon of generating an electric field due to the linear coupling between a specific material’s stress or strain state and its electrical polarization, which is inherently reversible. In 1880 Jacques and Pierre Curie observed the piezoelectric property of certain inorganic crystals. In the 1950s, lead zirconate titanate (PZT) and barium titanate (BaTiO_3_) were used for industrial and commercialization purposes for piezoelectric technology.^45^ Even though the piezoelectric property is prominent in ceramic crystalline materials, it is also inherent to some polymer materials such as ferroelectric polymers (PVDF), PA, polypeptides, and polyesters. The electric charge generated per unit area (in C) as a result of applied mechanical force (in N) is recognized as the piezoelectric coefficient, *d*_*ij*_, of piezoelectric materials, where *i* is the direction of electric field propagation and *j* is the direction of applied force.^45,136,137^ Sun et al. have derived equations from understanding the charge density, capacity, and maximum voltage resulting from the external force over piezoelectric nanostructures.^138^ Furthermore, Smith and Kar-Narayan have provided detailed information on the symmetry requirements, underlying mechanism, and further processing related to piezoelectric materials in ref (139). However, a theoretical understanding of the piezoelectric material is an essential factor in optimizing the PENG concept. Wang has derived equations using Maxwell’s equations in to calculate to output power through an external load(P) and total output energy (*E*_0_) of PENG devises as given below.^140^
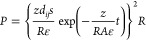
5
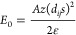
6

In eqs 5 and 6, *z* is the thickness, *s* is the strain, *R* is the external load, *A* is the surface area, *t* is the time, and ε is the permittivity of the material. Based on the equations, an increase of *d*_*ij*_ can drastically enhance the power output of PENG devises. In addition, Xu et al. provided evidence that surface nanostructures can distribute the piezoelectric potential over the cross section of the surface, thus being favorable for the final outcome.^141^ In wearable applications, polymer materials are favorable due to the mix of crystalline and amorphous regions. However, in comparison to ceramic materials which have a *d*_*ij*_ of 500 pC N^–1^, the initial polymer material’s coefficient is around 1/20th that of popular ceramic materials.^139^ Interestingly, electrospun PVDF nanofibers have resulted in *d*_*3*3_ of 57.6 pC N^–1^, while PVDF film exhibits 15 pC N^–1^, showing a significant improvement by electrospinning.^45^ Even though PVDF material has α, β, γ, and δ phases, the β phase is the most prominent phase with the highest spontaneous polarization yielding high piezoelectric power outputs.^82,142^ Electrospinning can be used as a poling mechanism to improve the β phase content, resulting in higher power output.^82,94,139^

The most prominent polymers used to create piezoelectric active layers for wearable applications include PVDF and its copolymers, PAN, cellulose and PLA, which are compatible with electrospinning, and can therefore provide a balance between electrical and wearable performance. Additionally, a high-voltage field in the electrospinning process can further enhance and adjust the electric poling of PENG materials. Mirjalali et al. have comprehensively reviewed electrospun PENG for energy harvesting and self-powered sensing,^94^ and Yu et al. have provided a review on electrospun organic nanofibers for bio applications.^45^ In this section, foremost consideration is given to developing PENG with electrospun nanofibers and composite PENG structures with electrospinning. We discuss the advantage of the electrospinning approach and how the device performance can be further improved with chemical and mechanical modifications.

Previous research on piezoelectric, pyroelectric, and ferroelectric materials suggested that the β phase with all-trans conformation with a dihedral angle of 180° of PVDF and its copolymers shows the highest output performance. He et al. have reviewed the effect of the different electrospinning parameters on tuning the β phase and crystallinity, thus increasing the piezoelectric performance of electrospun PVDF nanofibers. An increased applied voltage between the needle tip and collector can increase the number of charges and a higher degree of molecular orientation favorable for the crystallinity of PVDF nanofibers. However, increasing beyond 20 kV can accelerate the flying time of the charger without controlling their orientation, which results in a reduction of the piezoelectric properties of the material. Furthermore, TCD has a positive linear relationship between the formations of the β phase in the 9–15 cm region, beyond which it is seen to decline. It is not clear whether the behavior of the flow rate affects the piezoelectric performance; however, there is evidence that up to 2 mL/h increased flow rate increases the piezoelectric characteristic, with a decrease observed beyond this. Moreover, using a rotary collector and increasing the rotation speed up to 1500 rpm has increased the β phase and crystallinity. In addition to process parameters, solution parameters can be adapted to adjust the β phase and crystallinity. Such parameters include molecular weight (maximum output at 777000 g mol^–1^), concentration (the lower the better, but optimum is around 16–20%), and solvent volatility ratio (moderate volatile content is required; e.g. acetone 40%). Environmental conditions also affect the characteristics; e.g., maintaining a high-humidity environment at 25 °C temperature can increase the percentage of β phase (F(β)) in PVDF material.^82^

**Figure 7:**
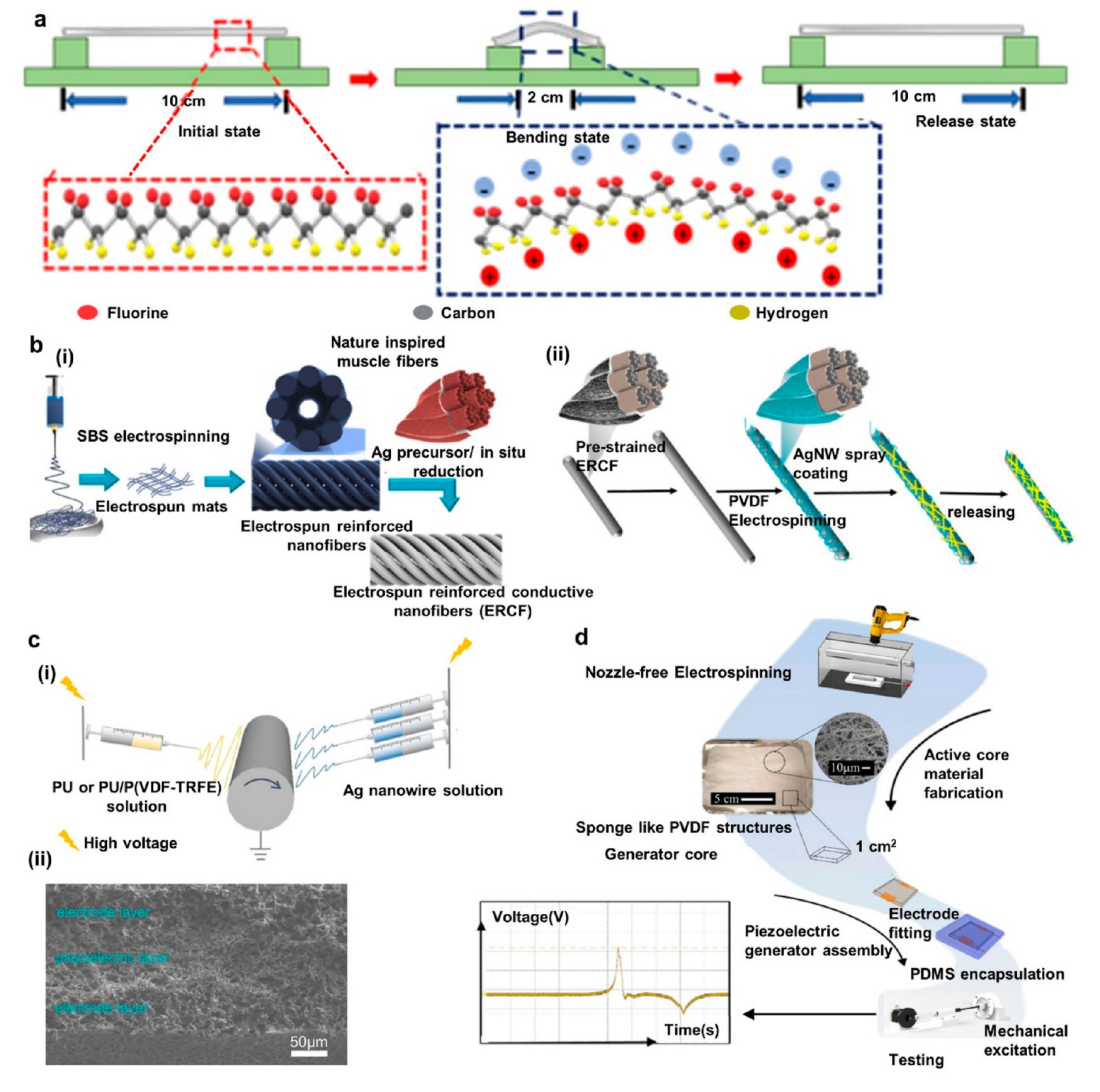
Electrospinning modified PENG based energy harvesting devices. (a) Schematic of MWCNT-loaded PENG device electrical performance characterization. Reprinted with permission under a Creative Commons [CC BY] License from ref (143). Copyright 2021 The Authors. Published by Elsevier Ltd. (b) Human muscle inspired electrospun conductive and piezoelectric layer based TENG device: electrode preparation process (i) and schematic of smart textile fabrication (ii). Reprinted from ref (144) with permission. Copyright 2022 Elsevier Ltd. (c) Composite electrospun PENG device with PU/AgNW and PU/P(VDF-TRFE): simultaneous electrospinning of PU and Ag nanowire (i) and SEM images of final device (ii). Reprinted from ref (145) with permission. Copyright 2021 IOP Publishing. (d) Needleless electrospinning to produce a 3D sponge structure. Reprinted with permission under a Creative Commons [CC BY] License from ref (146). Copyright 2022 The Authors. Published by Elsevier Ltd.

Recent advancements in PENG devices mostly use composite materials in electrospinning precursors to increase the power output of PENG devices. For example, Eun et al. added multiwalled carbon nanotubes (MWCNTs) to the electrospun precursor and observed tensile and piezoelectric performance. Electrospun fibers were oriented using a bespoke linear conveyor-based collection mechanism, increasing the β phase, tenacity, and initial modules of elasticity. The increase of MWCNT up to 0.008 wt % can increase *F*(β) by 46%, with a tenacity of 0.70 ± 0.01 g/d and initial modulus of 1.76 ± 0.19 g/d. These results are based on tensile testing carried out with the ASTM D2256 standard with 250 mm gauge length and 300 mm/min crosshead speed. It was observed that, on increasing the MWCNT percentage beyond 0.008 wt %, the performance was reduced. While the sample was attached to a piezoelectric tester (Figure 7a), a 0.01 wt % MWCNT sample resulted in *V*_OC_ of 0.71 V, which is a 343% increase compared with pristine randomly oriented PVDF nanofibers.^143^

Mimicking natural structures for technological development has been one of the most investigated areas by scientists for thousands of years. Among those, human biological-inspired devices are sometimes used in wearable electronic developments to address comfortability for wearers. Moreover, in PENG energy harvesting, the piezoelectric substrate and the conductive surfaces should process the balance of wearable and electrical performance at the same time. Considering these factors, Veeramuthu et al. have developed a human muscle-fiber-inspired conductive substrate using electrospun nanostructures (Figure 7b). Initially, a conductive substrate was prepared, reducing silver trifluoroacetate to form AgNPs on mechanically twisted 12 wt % elastomeric styrene–butadiene–styrene (SBS) electrospun substrate. Second, 16 wt % PVDF was electrospun on a 30% prestretched conductive substrate prepared in the early stage. Finally, AgNWs were spray-coated on the PVDF substrate for 75 s. The final design had excellent mechanical characteristics (elongation of 711.85%, toughness of 10.05 MJ m^–3^, and Δ*R*/*R*_0_ of 1.05 after 6000 cycles) and electrical characteristics (*V*_OC_ of 29.5 V, *I*_SC_ of 0.39 μA, and 11.57 μW power). The developed yarn using the electrospinning technique has 10-fold higher toughness than that for wet spinning techniques, providing promising results under stress and strain in human physical movements. The enhancement of the loading of conductive percolative networks aligns with Fick’s law of diffusion. This would indicate that the absorption of precursors and the quick formation of AgNPs occurring in electrospun samples are favorable for higher mechanical properties. The final design was successfully demonstrated in a self-powered smart glove for gesture recognition.^144^

Using suitable materials, the electrospinning technique can be used to develop highly conductive, water vapor permeable, air permeable composite structures for PENG architectures. Xue et al. have designed a PENG device with PU/AgNW electrospun electrodes and PU/P(VDF-TRFE) as a functional piezoelectric layer. In the composite structure, layer 1 was fabricated using a PU precursor and an AgNW ethanol dispersion. PU was electrospun and simultaneously AgNW was electrosprayed to the PU layer. Layer 2 was prepared using PU/P(VDF-TRFE) electrospinning, while layer 3 was prepared by repeating the procedure for layer 1 (Figure 7c). The developed electrode resulted in 1.4 Ω sq^–1^ sheet resistance while applying tensile strain up to 40% increased resistance up to 23.2 Ω sq^–1^. The composite structure had high water vapor permeability under a constructed test method due to the porous nature of the electrospun membrane. A repeated (20 Hz) force with 32 N applied over the device (3 cm × 4 cm) could generate *V*_OC_ of 47.9 V, *I*_SC_ of 31.8 μA, and a maximum power density of 35.3 μW cm^–3^ through a 10 MΩ resistor and was stable for 20000 cycles.^145^

Mechanical and chemical modification of the electrospun piezoelectric and conductive layers can significantly improve performance. For example, Diaz Sanchez et al. used needleless electrospinning technology to produce a sponge-like 3D structured PENG piezoelectric layer with LiCl salt loaded P(VDF-TRFE)/poly(ethylene oxide) (Figure 7d). Compared with conventional electrospinning this technique could produce nanofiber mats efficiently with thicker nanofibers. XRD analysis confirmed that a higher β phase is available within the developed sample compared with pristine PVDF. Furthermore, a 700 μm thick 3D structure sandwiched with copper foil when subjected to 1.58 N repeating (4 Hz) impact force has resulted in *V*_OC_ of 69.4 V and 40.7 μW cm^–2^ power through a 15.1 MΩ load resistor. Moreover, the device attached to a flip flop and walking 100 steps could charge a 1 μF capacitor to 15.31 V in 1 min.^146^

### 3.2 Electrospinning-Based Mechanical Self-Powered Sensing

The use of electrospun TENG and PENG devices as energy harvesters was discussed in sections 3.1.1 and 3.1.2, respectively. In addition to energy harvesting, the ability to generate charge from mechanical movements means that TENGs and PENGs can also be configured as self-powered sensing devices.

#### 3.2.1 Electrospinning-Based Triboelectric Self-Powered Sensing

A number of studies have developed sensors to monitor physiological signals from the human body, both *in vivo* and *in vitro* using electrospun membranes. The advantage of not relying on a separate power supply means that self-powered sensing with TENGs has been a highly investigated area in the field of wearable electronics.^1,136^ Alagumalai et al. have provided a comprehensive review on the possibility of combining machine learning and self-powered sensing, providing more pathways for future research.^147^ In the rest of this section, some examples of electrospun wearable TENG self-powered sensors will be discussed in detail.

**Figure 8:**
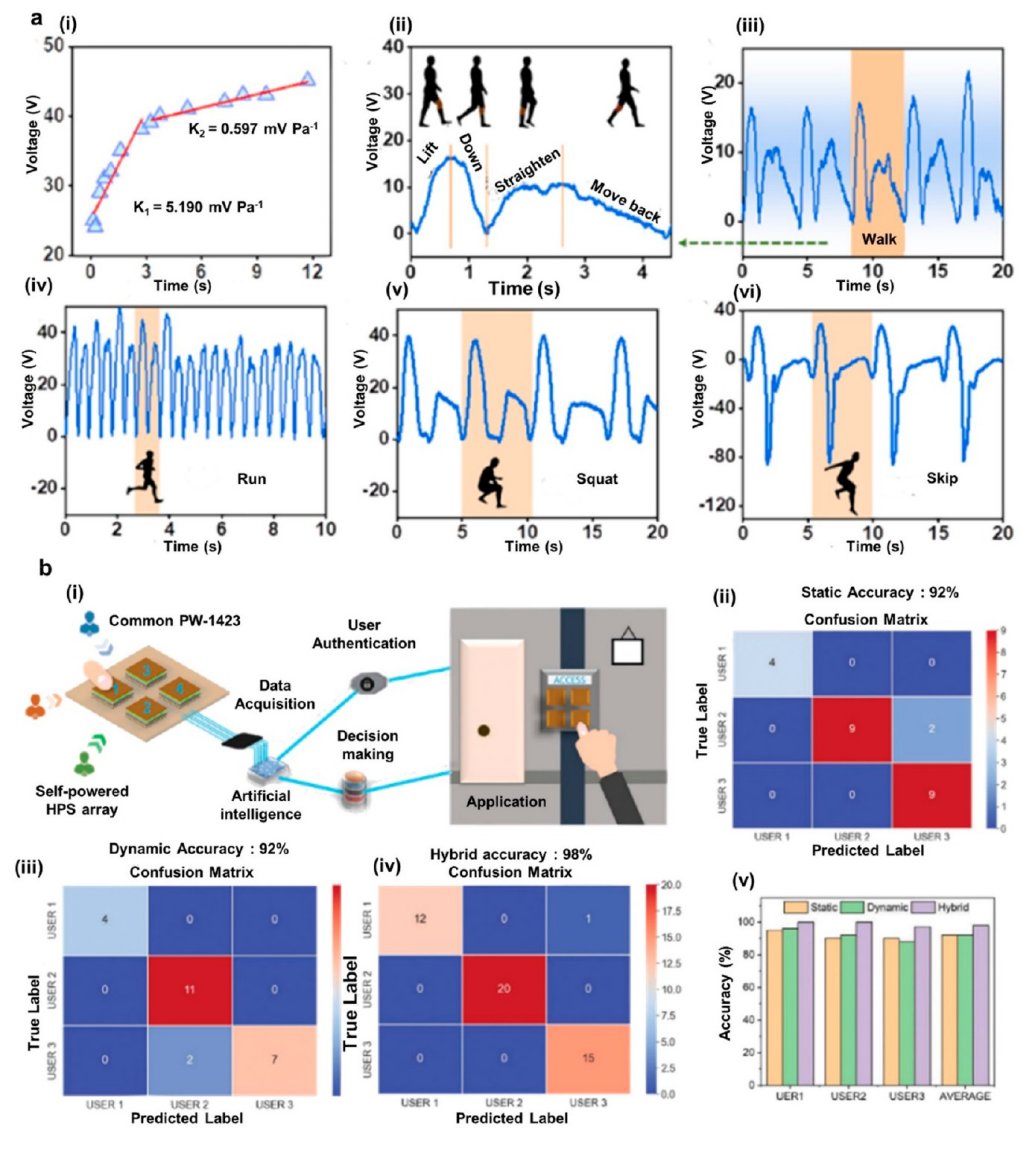
Applications of electrospinning-modified TENG-based self-powered sensing. (a) UV-protective, self-cleaning, and antibacterial nanofiber-based TENG sensor: output voltage vs applied pressure (i), different leg actions and corresponding results of the device (ii), and sensitivity with walking (iii), running (iv), squatting (v) and skipping (vi). Reprinted from ref (148) with permission. Copyright 2021 American Chemical Society. (b) Neural network based self-authentication system: schematic and architecture of the system (i), accuracy matrix for capacitive-based pressure sensor (ii), accuracy matrix for TENG-based pressure sensor (iii), accuracy matrix for hybrid pressure sensor (iv), and comparison of static, dynamic, and hybrid techniques (v). Reprinted from ref (29) with permission. Copyright 2022 Wiley-VCH GmbH.

Jian et al. have developed a TENG sensor for detecting human biomechanics using a TiO_2_@PAN electrospun membrane and nylon film as tribonegative and tribopositive layers, respectively, and a AgNW/TPU composite electrospun layer as the electrode. In addition to these layers, a polytetrafluoroethylene sandwich layer is used between the electrospun electrode and the tribonegative membrane to safeguard the electrode from moisture. The uppermost layer containing the TiO_2_ nanoparticles can absorb ultraviolet (UV) light and act as a self-cleaning, antibacterial agent. Furthermore, electrospinning is a highly efficient production method and provides a homogeneous nature for TiO_2_ over the PAN network, which is quite challenging with other fabrication methods. The device had a sensitivity of 5.2 mV Pa^–1^ in the region of 0–4 kPa and 0.6 mV Pa^–1^ for pressure >4 kPa. Furthermore, the device could detect and distinguish human motions such as walking, running, squatting, and skipping, as shown in Figure 8a. Moreover, the device could detect signals required for a self-powered pedometer system.^148^

Security is becoming one of the significant areas of focus worldwide due to the advancement of technology. Pressure-sensing-based user authentication is highly desirable in intelligent home and appliance control systems. The advance of smart devices and wearable technologies for authentication signals indicate the potential of embedding such functionality into sophisticated garments. Primarily static pressure sensing may be achieved through capacitive, resistive, and piezoresistive means, while dynamic pressure sensing information such as press time and hold time is challenging to measure with those techniques. Advanced approaches are needed to address these challenges in the future. One approach investigated by Bhatta et al. uses hybridized composite nanofiber-based TENG and capacitive pressure sensors to measure dynamic and static pressure. In this experiment, siloxane and PVDF are used in the same precursor to produce an electrospun tribonegative layer, while a nylon-66 electrospun layer is used as a tribopositive layer. High charge density propagation due to siloxane and high surface area due to electrospinning have provided circumstances to miniaturize the sensor up to the standard sensor size of 5 mm × 5 mm and hybridize with the capacitive technique with a maximum sensitivity of 12.062 kPa^–1^ in 0–3.5 kPa region and 2.58 V kPa^–1^ in the 3.5–25 kPa range. The rectified output of the TENG can be used to charge a capacitor in a self-powered approach. Finally, the sensor integrated into an AI system can predict the user with 98% accuracy (Figure 8b).^29^

#### 3.2.2 Electrospinning-Based Piezoelectric Self-Powered Sensing

Basic human activities such as walking, jumping, squatting, joint bending, joint rotation, muscle movements, and eye movements, as well as physiological measurements such as heartbeat, artery pressure, and respiration, are vital to monitor health and human lifestyle.^149^ This section will provide information on self-powered sensing using PENG and further enhancement of those devices’ sensitivity using the electrospinning technique.

**Figure 9:**
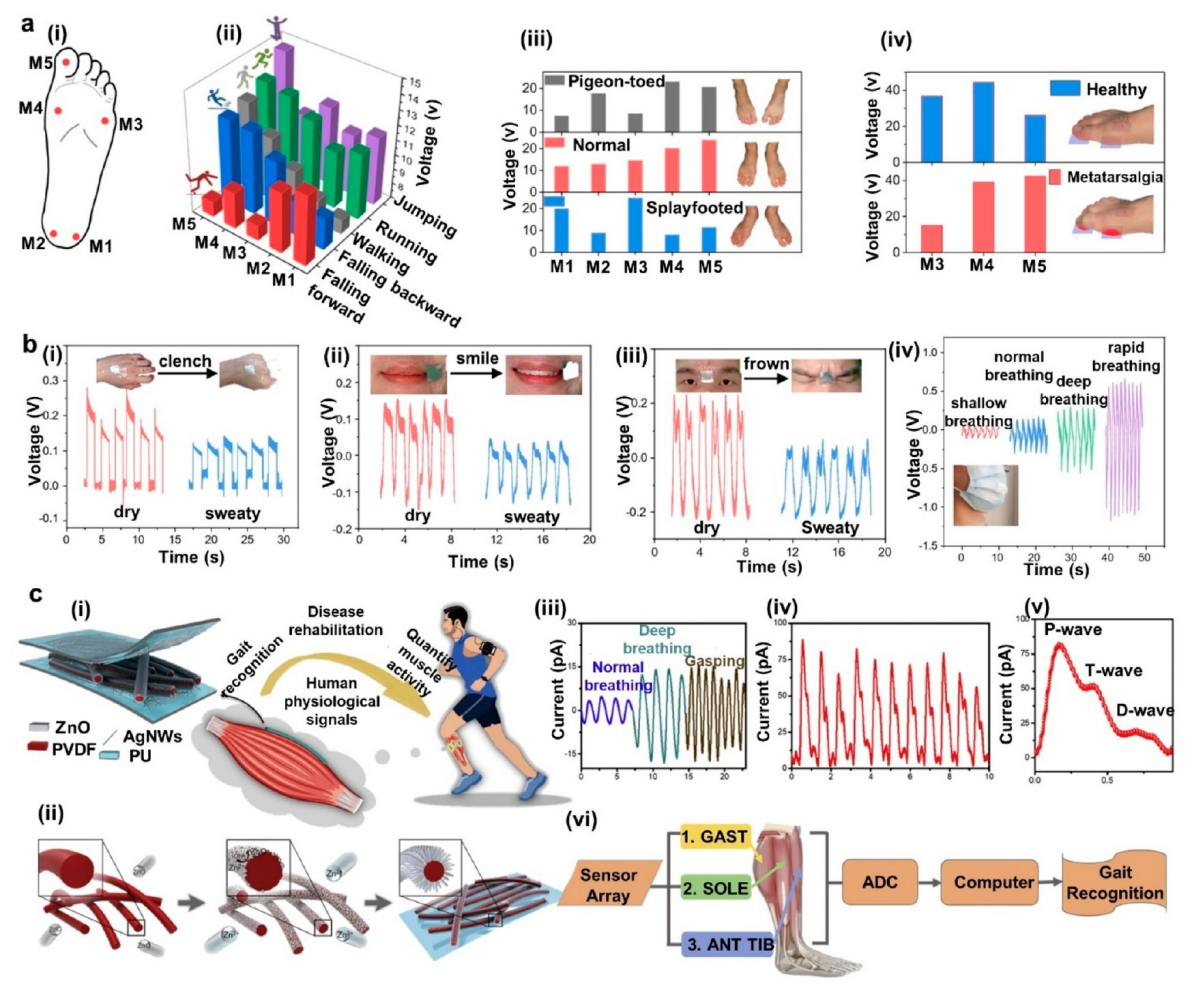
Electrospinning-modified PENG-based self-powered sensors to detect human motions and physiological signals. (a) High-performance piezoelectric-composite-based PENG sensor: schematic of integration in smart insole (i) and identification of gait monitoring (ii), posture abnormalities (iii), and metatarsalgia complication prognosis (iv). Reprinted with permission under a Creative Commons [CC BY] License from ref (150). Copyright 2022 The Authors. Published by Springer Nature (b) Sensing–transducing coupled piezoelectric textiles for real-time monitoring: clench (i), smile (ii), frown (iii), and breath monitoring (iv). Reprinted from ref (151) with permission. Copyright 2023 Royal Society of Chemistry. (c) Electrospun PVDF/ZnO core–shell nanofiber based PENG: schematic and muscle behavior monitoring (i), fabrication process (ii), breath monitoring (iii), wrist pulse monitoring (iv), the expanded pulse determining the 3 peaks (v), and schematic of developed sensor system (vi). Reprinted from ref (152) with permission. Copyright 2020 Elsevier Ltd.

An example of such a device was developed by Su et al., where a PENG-based self-powered sensor was used for gait pattern monitoring, identification of walking habits, and determining metatarsalgia complications. In device fabrication, samarium-doped lead magnesium niobate/lead titanate based PVDF (Sm-PMN-PT/PVDF) was used to produce the electrospinning precursor. 2.5 wt % loading of Ti_3_C_2_T_*x*_ (MXene lamellae) to the precursor increased the piezoelectricity of Sm-PMN-PT/PVDF by 160%. XRD spectrometry on a sample provides evidence that the final sample has a polycrystalline perovskite structure and at 2.5% load of MXene has provided the highest peak related to β phase. The incorporation of MXene at the precursor has minimal impact on the morphology of the electrospun sample, thus providing favorable wearable characteristics. Increasing applied force from 1 to 9 N increased the voltage and current from ∼7 to ∼12 V and ∼0.25 to ∼1.2 μA, respectively. The developed sensor was attached to a shoe insole in five different positions, as depicted in Figure 9a (i). Interestingly, there was a distinguished difference in signal output related to jumping, walking, running, falling backward, and falling forward (Figure 9a (ii)). In addition, the sensor system could detect posture abnormalities such as pigeon-toed or splay-footed (Figure 9a (iii)) and can be used for the clinical prognosis of metatarsalgia conditions (Figure 9a (iv)).^150^

Moisture content due to environmental factors such as humidity and temperature can drastically reduce the sensitivity and transduction of PENG-based wearable sensors. In addition, fluoropolymers have weaknesses such as a weak output signal (*d*_33_ = 29 pC N^–1^) and poor detection limitations (100 ppm), which are not favorable toward signal transduction in humid environments. Su et al. developed a humidity-compatible wearable biomonitoring sensor by introducing poly(ether imide) (humidity sensing material) to a samarium-doped PMN-PT (*d*_33_ = 1500 pC N^–1^) electrospinning solution. Electrospun fibers had an average diameter of 460 nm with samarium-doped PMN-PT 100 nm nanoparticles. The naturally porous structure was enhanced with poly(ether imide) to facilitate chemisorption of moisture particles efficiently and effectively. The final sensor resulted in a sensitivity of 5.7 V N^–1^ with a linearity of 0.986 (*R*^2^). In addition, the performance was slightly affected during small humidity changes at low humidity, i.e. from 0% to 0.9%, but drastically affected by large humidity changes from 7% to 97.3% RH, which could be recovered within 20 s. It was demonstrated that the electrospinning time concurrently with the layer thickness have a proportional positive relationship with the piezoelectric coefficient and permittivity. Furthermore, the device could be used to detect facial expressions and static sweat-streaming levels related to anxiety. It was also used to continuously identify shallow, regular, deep, and rapid breathing patterns (Figure 9b).^151^

Yang et al. used PVDF/ZnO to develop a superfine coaxial hierarchically structured PENG sensor for monitoring physiological signals such as respiration, wrist pulse, and muscle behaviors. The fabrication process was conducted in three steps: initially electrospinning of PVDF, then conformally coating ZnO nanocrystals using magnetron sputtering over electrospun surface, and finally epitaxially and coaxially growing ZnO nanorods using zinc cations and oxygen anions (Figure 9c (i, ii)). High surface area and porous structure due to the electrospinning process provide excellent compatibility, high surface contact with human skin, and high gas/air permeability. The prototype contains PVDF nanofibers of 800 nm diameter and 25–50 nm ZnO nanorods with a typical thickness of 35 μm. The PVDF/ZnO nanofiber-based device had a sensitivity of 3.12 mV/kPa, while PVDF fibers had a sensitivity of 0.527 mV/kPa. The final device successfully identified normal, deep, and gasping breath patterns (Figure 9c (iii)). Furthermore, it was sensitive enough to identify the wrist pulse (Figure 9c (iv)) and could distinguish percussion, tidal, and diastolic waves related to the period of a pulse (Figure 9c (v)). In addition, attaching the device to the epidermis of the calf muscle along with suitable circuitry was used for gait monitoring.^152^

Assistive communication technologies are an essential feature for people with health conditions or impairments. Lee et al. developed a motion communication method using a P(VDF-TRFE) electrospun nanofiber-based PENG device. The electrospinning technique can be used to miniaturize the sensors and reduce the dielectric constant by trapping air in a porous structure favoring higher sensitivity. During the experiment, surface porosity was controlled by non-solvent-induced phase separation, kinetics related to electrospinning, and thermodynamic properties of P(VDF-TRFE) polymer. Interestingly, an increase in surface porosity notably outperformed the nonporous counterparts in terms of power outputs and sensitivity. The final device could successfully demonstrate real-time motion to display a communication method targeting wearable and robotic applications.^153^

### 3.3 Solar-Based Harvesters

Harvesting energy from sunlight using the photoelectric effect is another sustainable approach to generating clean energy. The photoelectric effect occurs by irradiating sunlight or other suitable light source upon a semiconductor device that results in the release of sufficient free electrons to generate current in an external circuit. This is the fundamental mechanism of solar energy harvesting (SEHG).^12^ Characterization of such SEHG devices can be described by their fill factor (FF) and power conversion efficiency (PCE). The FF provides insight into the performance of the SEHG device at the point of maximum power drawn. In addition, PCE provides information regarding the amount of usable power converted from the solar power input.^154,155^ Third-generation photovoltaic techniques, namely DSSC,^49,156,157^ PSC,^10^ and OSC,^15^ are prominent in wearable electronic applications. For the fabrication of parts of the DSSC, OSC, and PSC SEHG device structures, electrospinning has many advantages. Blachowicz and Ehrmann have provided a comprehensive review on the optical properties that can be enhanced by using electrospun nanofibers due to high surface area and natural pore structure.^158^

**Figure 10:**
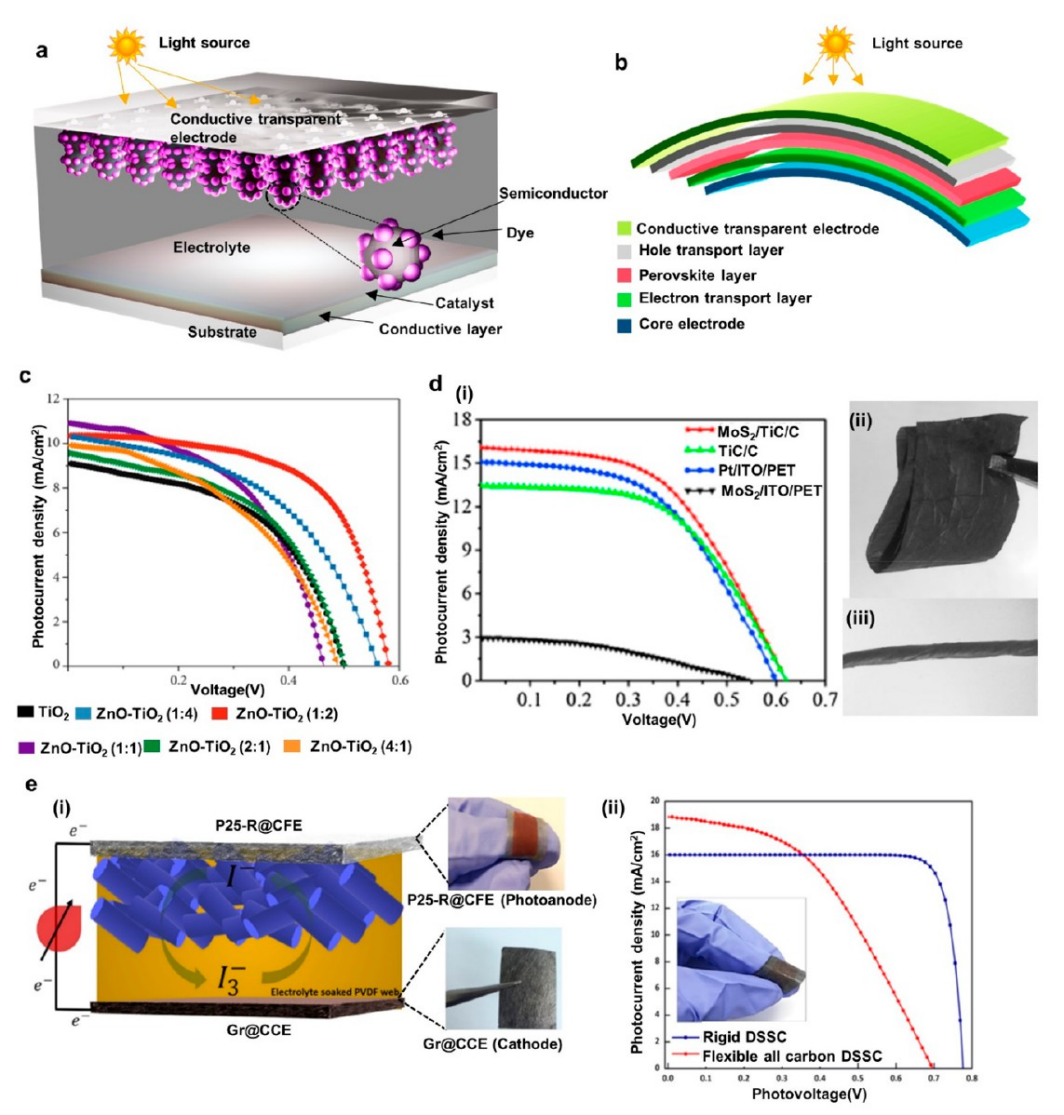
Electrospinning modified solar energy harvesting devices and DSSC examples. (a) Schematic of DSSC adapted from ref (165) with permission. Copyright 2022 The Author(s), under exclusive license to Springer-Verlag GmbH Germany, part of Springer Nature. (b) Schematic of PSC. Adapted from ref (10) with permission. Copyright 2020, Royal Society of Chemistry. (c) Current density and voltage plot for ZnO- and TiO_2_-based DSSC devices reprinted with permission under a Creative Commons [CC BY] License from ref (163). Copyright 2022 The Authors. Published by Hindawi Publishing Corporation. (d) Current density and voltage graph of developed photovoltaic devices (i), folded (ii) and twisted (iii) to form yarn images of MoS_2_/TiC/C nanofibers film. Reprinted from ref (156) with permission. Copyright 2019 Elsevier Ltd. (e) Schematic (i) and current density and voltage plot (ii) of electrospun PVDF-HFP based DSSC. Reprinted from ref (166) with permission. Copyright 2020 Elsevier Inc.

Among third-generation photovoltaic techniques, DSSC has gained significant attention toward wearable applications due to excellent flexibility, low cost of fabrication, ease of manufacturing process, and affluence of materials to develop devices. DSSC was reported in 1991 and resulted in 14.3% PCE under the standard air mass of 1.5 global conditions in 2015 with low cost and simple fabrication methods.^51^ A typical DSSC consists of flexible electrodes (one of which needs to be transparent), an electrolyte, and a photoactive layer with a specific dye material^159^ (Figure 10a). Even though DSSC is popular due to the previously mentioned factors, there are significant drawbacks, as there is low power conversion efficiency in practical scenarios due to low mechanical stability and a complicated sealing process. On the other hand, electrospinning provides promising results for DSSC in areas such as flexible semitransparent electrode development,^49^ highly flexible, biocompatible, eco-friendly, highly conductive electrode development, and semi-solid-state electrolyte layer development.^95,156^

In addition, Tang developed a successful OSC in 1985. Since then material research has increased the PCE of such devices from 1% to 14.4%.^15,160^ Typically, OSC devices have a thickness range of 10–100 μm, making them suitable for wearable applications.^160^ Interestingly, OSC devices can be produced with biodegradable, sustainable materials with properties such as lightweight, transparency, softness, and low cost.^161^ The architecture of OSC devices is more or less similar to those of DSSC devices, replacing the middle layer with an organic polymer active layer.^160,161^ Even though in the early days electrospinning has been used to develop organic solar cells, it is challenging to find recent developments with this technique.^89,98^

PSC is a commercially viable energy conversion method among other third-generation techniques. Furthermore, PSC has a reported PCE of ∼25.7% in flat surfaces and 15.7% in fiber/yarn architecture.^10^ Typical perovskite materials mainly follow or contain the crystalline structure of calcium titanium oxide (CaTiO_3_) with 1.5–2.5 eV band gap, 10^5^ absorption coefficient, 800 cm^2^/(V s) carrier mobility, and 10^10^ cm^3^ (single crystals)/10^15^–10^17^ cm^3^ (polycrystalline) trap-state density.^162^ The traditional PSC has the DSSC architecture with a perovskite layer sandwiched between the electron and hole transport layers and their attached electrodes (one transparent) (Figure 10b). Furthermore, solid-state mesoscopic, meso-superstructure, planar n-i-p heterojunction, and inverted planar structures are prominent architectures among the scientific community in PSC-related research. Balilonda et al. have provided a comprehensive review on fiber-shaped perovskite devices which can be used in wearable applications.^10^ Due to the perovskite material’s composite nature, it can be embedded into garments as yarns using a single-step electrospinning process.^10,33^ Wearable SEHG is prominent with techniques such as DSSC and OSC and most widely with PSC devices. Electrospinning can be used to develop photoanodes, CEs, and electrolyte layers of wearable SEHG targeting higher outputs. The next section of the review will cover some examples that have used the electrospinning technique to enhance the power output while retaining wearable characteristics.

#### 3.3.1 Electrospinning Based DSSC Devices

Wearable DSSC devices must be flexible enough to retain functioning under rigorous bending and stretching motions. TiO_2_ is a commonly used wearable photoanode semiconductive material due to biocompatibility, environmental friendliness, cost-effectiveness, and stability. However, traditionally coated TiO_2_’s low specific surface area limited the carrier transmission rate. Adding ZnO followed by nanofabrication techniques sophisticatedly resulted in a high PCE compared with TiO_2_ coatings. Chang et al. successfully increased PCE by 56%, resulting in a maximum of 3.66% using electrospun ZnO-TiO_2_ (1:2 molar ratio) composite nanofibers compared with TiO_2_ nanofibers. SEM analyses and BET multipoint methods provide evidence of ultrafine fiber morphology resulting from the electrospinning process with a large specific surface area. Additionally, evenly distributed tiny pores due to the electrospinning process have increased the charge transport rate, resulting in higher outputs. The final device exhibited a *V*_OC_ of 0.58 V, a current density of 10.36 mA cm^–2^, and a FF of 0.61 (Figure 10c).^163^ Moreover, Nien et al. observed that Fe_2_O_3_/TiO_2_ and g-C_3_N_4_/TiO_2_ electrospinning with a double jet to produce a heterogeneous nanofiber composite had improved the PCE up to 4.81%. The utilization of the double jet electrospinning method to produce heterogeneous fibers has enhanced the scattering effect, leading to a higher light collection and ultimately increasing the number of photoelectrons.^164^

In DSSC developments, Pt or transparent conductive inks have been used as CEs. However, in wearable DSSC applications, it is required to have highly flexible, excellent catalytically active and high-transmittance CEs to facilitate natural body movements and reduce the backlight illumination. Zhou et al. developed a flexible CE with Pt by the electrospinning technique and achieved 80–85% transmittance while maintaining sheet resistance of 100–150 Ω sq^–1^. During this experiment, PVA was electrospun as a sacrificial layer, and Pt was magnetron sputtered on the PVA layer followed by PVA dissolved using DI water. Interestingly, the developed Pt network sheet resistance has not increased significantly for 1000 bending cycles while the sheet resistance of ITO/PET has increased by 6 times. In addition, the DSSC developed with Pt nanofibers (sheet resistance 130.2 Ω sq^–1^ and 85% transmittance) has resulted in a *V*_OC_ of 0.68 V, a current density of 10.99 mA cm^–1^, a PCE of 3.82%, and a FF of 0.53, and 90% of the output was retained after 200 bending cycles.^49^

Even though transparent conductive inks provide high conductivity, using them for flexible substrates is challenging due to their brittle nature. On the other hand, Pt is an expensive and scarce noble material, providing additional issues related to practical applications and scalability. Considering these factors, Song et al. have used a TiC/C electrospun nanofiber film as the CE for the DSSC application. Photocurrent density–voltage measurements (Figure 10d) provide the highest results for MoS_2_-modified TiC/C electrodes, while the Pt/ITO/PET substrate shows a lower output. The initial PCE of the device was 5.08%, which slightly decreases after 100 cycles, indicating the device possesses some mechanical stability.^156^ In another example, Wu et al. used boron-doped electrospun CNF to produce a DSSC device resulting in a PCE of 7.51%. In addition, the developed CE layer possesses improved wettability, high catalytic activity, and high charge transfer properties, making it suitable for use as a shared electrode for supercapacitors and DSSC self-powered applications.^167^ These experiments suggest the possibility of integrating different materials as electrospun nanofibers for CEs in DSSC devices for higher output with possible commercially viable scalable products.

Electrospinning can produce very thin separation layers with different functionalities. In DSSC applications, it is essential to maintain sufficient distance between electrodes to prevent short circuits of the cell. At the same time, the device architecture must have a low thickness to facilitate wearable performance. Arbab et al. used the PVDF-HFP electrospun sample as the spacer fabric between the electrodes. Using the electrospinning technique, the layer thickness was maintained at 10 μm, and electrolyte was injected into the electrospun layer after carefully sealing (Figure 10e (i)). Developed DSSC had PCE of 5.92% and FF of 45.109% with *V*_OC_ of 0.696 V and current density of 18.838 mA cm^–2^. Also, as given in Figure 10e (ii), the device had greater flexibility with a low thickness, making it suitable for wearable applications.^166^

Using liquid electrolytes for wearable DSSC applications is challenging due to leakage issues under rigorous bending movements. Some researchers have used solid-state or quasi-solid-state electrolytes to address this issue. When developing a quasi-solid-state electrolyte, it is vital to maintain a high amount of liquid electrolyte in the membrane for favorable ionic conductivity and superior ion diffusion while maintaining better interfacial contact. Thomas et al. have used phthaloyl agarose, poly(3-butyl-1-vinyl imidazolium iodide), conductive carbon, and PVA electrospun membrane to produce a quasi-solid-state electrolyte for DSSC application. The higher surface area due to the electrospun nanofiber nature has provided sufficient improvements for the performance of DSSC. Transmission electron microscopic images have revealed the successful incorporation of electrolyte materials on a PVA host polymer matrix. In addition, with a superporous structure, ionic conductivity has been recorded as 6.3 × 10^–3^ S cm^–1^. The final device exhibited a current density of 13.1 mA cm^–2^, a *V*_OC_ of 0.79 V, and a PCE of 6.05% with excellent stability after 500 h.^168^

#### 3.3.2 Electrospinning-Based OSC and PSC Devices

**Figure 11:**
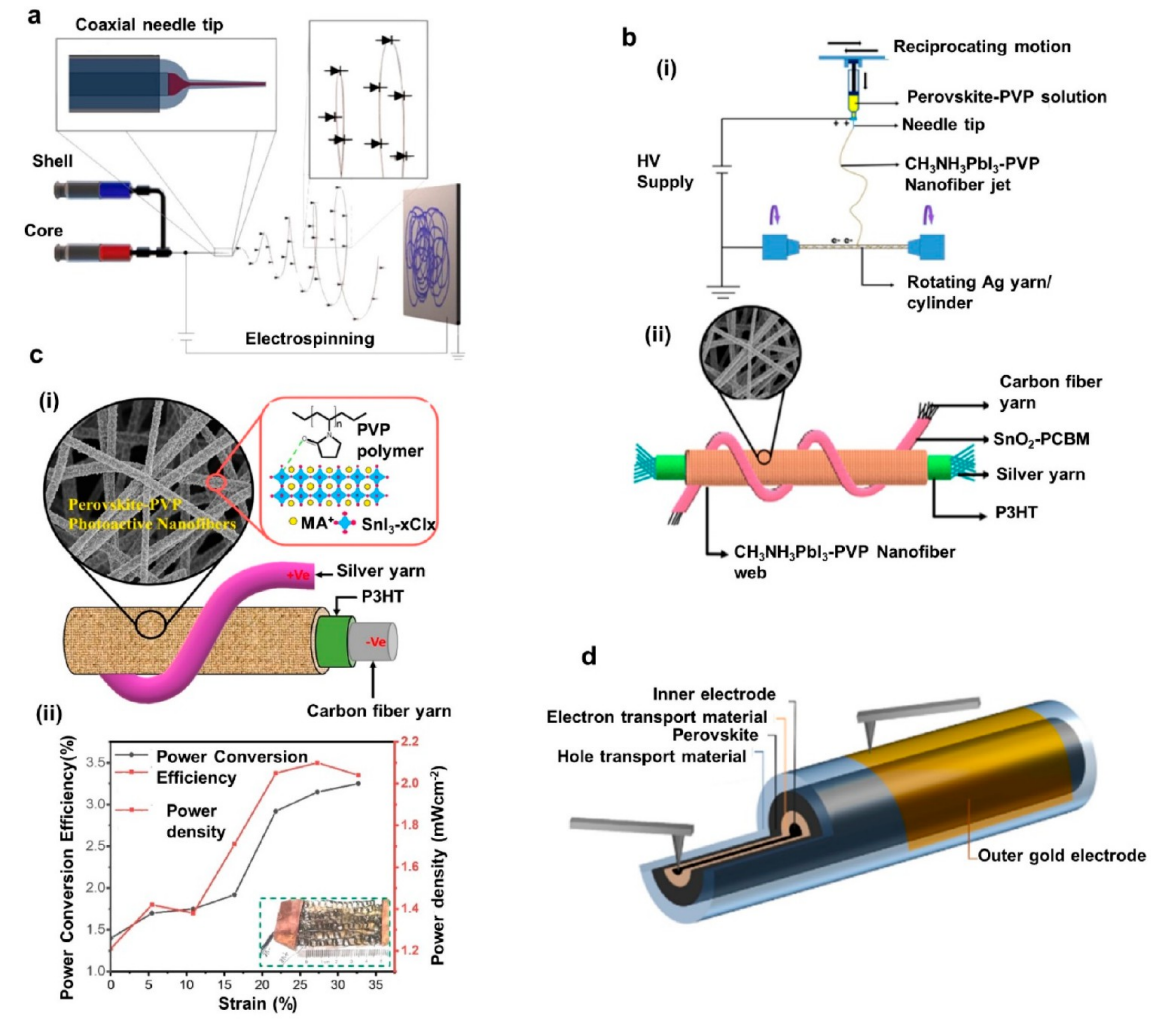
Electrospinning modified surface using OSC and PSC device examples. (a) Coaxial electrospinning technique to produce a p–n junction for solar cells. Reprinted with permission under a Creative Commons [CC BY] License from ref (89). Copyright 2022 The Authors. Published by MDPI. (b) Flexible solar yarn made with electrospinning process depicted: the fabrication schematic (ii) and final design architecture (ii). Reprinted from ref (99). Copyright 2020 Wiley-VCH Verlag GmbH & Co. (c) Lead-free perovskite yarn development for SEHG knitted fabric manufacturing: schematic of the developed yarn (i) and PCE and power density (ii) of solar yarn based knitted fabric. Reprinted from ref (169). Copyright 2020 Elsevier BV. (d) Triaxial nanofiber based perovskite solar cell schematic. Reprinted from ref (33). Copyright 2021 The Authors. Advanced Engineering Materials published by Wiley-VCH GmbH.

One-step electrospinning for bulk heterojunction organic photovoltaic devices has been examined, targeting flexible applications. Bedford et al. have used PCL as the sacrificial sheath material to produce a P3HT:PCBM-based OSC device using a coaxial electrospinning process. After the electrospinning sheath layer was dissolved, a P3HT:PCBM layer was deposited on a PEDOT:PSS electrode to develop the final device. There was a clear indication that using the electrospinning technique to produce nanofibers (PCE of 4.0%, FF of 63%, current density of 10.7 mA cm^–2^, and *V*_OC_ of 0.59 V) increased the performance compared with a film-based (PCE of 3.2%, FF of 54%, current density of 10 mA cm^–2^ and *V*_OC_ of 0.59 V) system. In addition, uniform phase separation with photovoltaic materials and avoiding issues with blending polymers can be achieved using the coaxial electrospinning technique.^98^ Recently, Serrano-Garcia et al. produced a p–n junction using P3HT as the core and poly(benzimidazobenzophenanthroline) (BBL) as the sheath using coaxial electrospinning (Figure 11a). Because of the nanofabrication nature of electrospinning, the developed yarn diameter could maintain 280 nm to 2.8 μm. These experiments provide pathways to produce nanoscale to microscale solar cells for future wearable applications.^89^

It is always preferable for wearable SEHG device electrodes to be flexible and transparent. Considering these factors, Cao et al. have developed nanofiber web electrodes by electrospinning phosphor/PI/PU@silver composite material. The final device had a sheet resistance of 22.1 Ω sq^–1^ and UV transmission of over 80%, providing suitable characteristics for wearable SEHG device electrodes. Furthermore, the developed electrode, along with ZnO, CH_3_NH_3_PbI_3_ and 2,2′,7,7′-tetrakis(*N*,*N*-di-*p*-methoxyphenylamine)-9,9′-spirobifluorene was used to manufacture a PSC device which resulted in a PCE of 3.47%. In addition, the developed PSC had good bending stability, and the electrode could be stretched up to 100%, retaining sheet resistance of 30.5 Ω sq^–1^ and 83.5% transmission. After 500 cycles stretching up to 50%, the sheet resistance increased by 75%.^100^

In recent history, one of the highly investigated research areas related to wearable SEHG has been the development of fibers/yarns and subsequently conversion of these into fabrics that exhibit a balanced wearable and electrical performance. Due to the solid nature of perovskite materials, the fabrication of those materials into yarns is much easier than for DSSC systems. Li et al. developed a highly flexible PSC with average weight (0.89 mg cm^–1^) and high active lifetime (>216 h) by electrospinning perovskite material (Figure 11b (i)). To create the anode part, an electrospinning solution was prepared, adding CH_3_NH_3_I and PbI_2_ (photoabsorber perovskite layer) into PVP (in anhydrous DMF), and electrospinning was carried out onto a rotating P3HT (organic hole conductive layer) coated silver yarn (Figure 11b (ii)). The grain size of the perovskite material was controlled by controlling the RH (75%), postheating process, and applied voltage (18 kV). The cathode was prepared by dip-coating carbon fiber yarn with magnetically agitated PCBM and electrospun SnO_2_ (electron conductive layer) nanofibers. Anode and cathode yarns were prepared separately and twisted together for the final device. The optimized twisted yarn (thickness 10.5 μm) could generate a current density of 11.94 mA cm^–3^, a *V*_OC_ of 1.92 V, a FF of 54.2% and a PCE of 15.7% that was retained after 750 bending cycles. In addition, the fabric prepared using these yarns could generate a power density of 1.26 mW m^–2^. It was shown that using the electrospinning technique, perovskite materials have been uniformly distributed, directly wrapped, and compactly assembled over the targeted substrates, resulting in higher bending and functional performance.^99^

In another study, Balilonda et al. have developed a lead-free perovskite yarn by electrospinning CH_3_NH_3_I mixed PVP doping [6,6]-phenyl C61 butyric acid methyl ester (PC_61_BM). This study targeted a knitted structure fabric-based wearable SEHG application (Figure 11c (i)). P3HT-coated carbon yarn was covered with an electrospinning solution with experimental parameters selected at RH (75%) and voltage (18 kV) to produce an anode for the final device. The developed yarn could absorb more than 90% of the optical band gap of 1.65 eV in the wavelength region of 300–550 nm, ensuring PCE of 7.49% by doping 0.17% PC_61_BM to the electrospun layer. Interestingly, the yarn structure could maintain 98% of the initial PCE after 1000 bending movements. Furthermore, yarn could be converted into knitted fabric with dimensions of 45 mm × 35 mm and could generate a maximum power output of 1.21 mW cm^–2^ under 1000 W m^–2^ solar illumination (Figure 11c (ii)).^169^

Advancement in a one-step electrospinning process can be used to fabricate photo absorber, hole, and electron transport materials in a concentric axial cable. Bohr et al. has created such devices to manufacture tiny solar cells that can be converted into fabrics suitable for wearable applications (Figure 11d). As a prototype development, they used coaxial and triaxial electrospinning processes to fabricate CuSCN/MAPbI_3_ and CuSCN/MAPbI_3_/ZnO-Zn(OAc)_2_ base systems, respectively. In the triaxial approach, CuSCN (hole transport material) is used as the core, while MAPbI_3_ (photoaborber perovskite) is used as the intermediate layer and ZnO (electron transport layer) as the shell. Further experiments need to be conducted to develop a fully working solar cell with electrospinning in the future.^33^

### 3.4 Electrospinning-Based Thermoelectric (TEG) and Moisture (MEG) Energy Generators

The natural phenomena of maintaining a human body core temperature at 37 °C and 60–180 W heat dissipation from the human body based on the activity level causes the use of thermoelectric energy harvesting concepts for powering wearable electronic applications. The discovery of the Seebeck effect in 1821 by Thomas Seebeck, followed by the Peltier effect in 1834 by Jean Peltier and the Seebeck voltage in 1851 by Gustav Magnus, were fundamentals that led to the development of the concept of TEGs.^14,170,171^ When two materials made of semiconductors or conductors with different electrical properties are connected directly or through a conductive path, a voltage called the Seebeck voltage can be generated due to the diffusion of charge carriers in the presence of a temperature gradient (from the high-temperature end to the low-temperature end).^171^ The dimensionless figure of merit (*zT*) is used to evaluate the thermoelectric potential of thermoelectric materials and can be calculated using eq 7
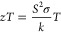
7where, *S* is the Seebeck coefficient, σ is the electrical conductivity (depends on carrier charges, mobility, and concentration), and *k* and *T* are the thermal conductivity (combination of lattice and electron thermal conductivity) and absolute temperature, respectively.^172^ Popular thermoelectric materials such as bismuth telluride combined with metal alloys are often rigid, expensive, and nonbiocompatible.^14,173^ Nozariasbmarz et al. have provided a comprehensive review on wearable thermoelectric energy harvesters, providing evidence that nanostructured (Bi_*x*_Sb_1–*x*_)_2_Te_3_ and Bi_2_Te_3–*x*_Se_*x*_ are mostly suitable for wearable TEG applications. The review also highlights the possibility of developing flexible TEGs using coating or printing techniques with either flexible or rigid interconnects for wearable applications.^14^ However, since the long-term efficiency of such TEG devices decreases due to wearing and abrading, Ewaldz et al. suggested that electrospinning is a better alternative. In addition to their flexibility and stretchability, materials produced through the electrospinning technique possess naturally high surface area and porosity, leading to significantly reduced thermal conductivity and ultimately higher *zT* values.^173^

TEG devices typically rely on p- and n-type material-based systems to generate high power in real-world applications. However, CNT stands out for its ability to convert carrier type compared to other materials. While CNT is traditionally prepared by a floating gas-phase catalysis technique, this method creates high thermal conductivity and is not ideal for TEG devices. An alternate wet spinning technique has been used, but it requires integration with additional insulating polymer as reinforcement, which can reduce TEG performance.^174^ To address these challenges, Jin et al. utilized an electrostatic spray technique to develop high-performance CNT-based TEG (26.2 nW at T of 33.4 K).^175^ He et al. utilized a coagulation bath electrospinning method to produce a TEG that is both stretchable and interactive, taking wearable TEG to the next level. The electrospinning precursor was a mixture of poly(ethylene imine) and PU, doped with PEDOT:PSS and loaded into a syringe. Flow rate, applied voltage, and TCD were adjusted to 0.5–1 mL/h, 8–12 kV, and 3–5 cm, respectively, and the resulting fibers were collected in a CNT/PEDOT PSS bath. The optimal CNT:(PEDOT:PSS) ratio in the coagulation bath was 4:6, resulting in excellent electrical conductivity and a Seebeck coefficient of 44 μV K^–1^. The coagulation bath electrospinning technique enabled self-assembly of CNT with even distribution over the nanofiber network, increasing conductivity and power factor by 10 times. Additionally, the yarn was highly flexible and stretchable, with a strain of 350%, making it easy to integrate into traditional clothing using simple sewing techniques for energy harvesting applications.^176^

Wearable energy harvesting techniques have been advancing rapidly, and MEG is one of the concepts that shows great potential. Electrospinning has been used as a fabrication technique to optimize these devices, making them more efficient and effective. Materials that are active and contain oxygen-functional groups, such as hydroxy (−OH), carboxyl (−COOH), and sulfonic acid (−SO_3_H), are capable of capturing moisture molecules when prompted by an environmental stimulus. Once these functional groups are ionized by the moisture, they can release free protons due to their asymmetric structure. This flow of protons from an area of high concentration to an area of low concentration creates a current flow, which serves as the foundation of MEG devices.^177,178^ In order to enhance MEG devices, it is necessary to improve the inner gradient structure of the hygroscopic material through techniques such as nano/microfabrication or chemical modifications.^179^ Zhang et al. found that the MEG device’s output is closely tied to its structural characteristics, including hydrophilicity, porosity, and specific surface area. To address these aspects, the authors have created a cost-effective wearable MEG device that uses tetrabutylammonium bromide (TBAB) mixed with CA electrospun nanofibers. Electrospinning TBAB into cellulose acetate creates a membrane with excellent hydrophilic properties (pristine electrospun CA—contact angle 132° reduced to 26° with the addition of 2% TBAB), enabling water molecule transport and ion migration. The surface area increases, resulting in better moisture absorption and higher output. The output can be further enhanced by decreasing pore size by changing the nanofiber diameter and increasing interwind structures. By controlling these parameters, the device’s output has improved from 110 mV (pristine CA) to 700 mV with a maximum power of 2.45 μW cm^–2^ at 90% relative humidity.^180^

In the literature to date, carbon-based materials like carbon black, CNT, and reduced graphene oxide are commonly utilized as electrodes for MEG devices because of their high conductivity, easy preparation, and widespread availability.^181^ However, these materials’ low flexibility and stretchability pose challenges for their use in wearable applications. To address this, Faramarzi et al. have developed a stretchable and flexible MEG utilizing electrospun polysulfone and PU materials. Combining these polymers in a 2:8 ratio results in a mixed polymer matrix that exhibits excellent stretchability, providing an effective substrate for layer-by-layer coating of MWCNT to create the final device. By controlling the pore structure and fiber mat thickness, the capillary flow of water may be regulated, resulting in higher output for the MEG device. The final device, measuring 1 cm × 2 cm, is capable of generating a *V*_OC_ of 419 mV and *I*_SC_ of 1.5 μA and can withstand stretching up to 60% without experiencing output degradation.^182^

### 3.5 Electrospinning-Enabled Wearable Energy Storages

In order to overcome the transient generation of energy with energy harvesting techniques, there is a need to develop wearable energy storage solutions that can ensure a continuous supply of power. In mechanical energy harvesting techniques, power/signal output is instantaneous and irregular, based on the amplitude, frequency, and force of the external mechanical stimuli.^114,183^ SEHG devices require the presence of sun or light source radiation to generate electricity continuously. Energy storage devices need to be combined with energy harvesting systems. Supercapacitors and rechargeable flexible Li-ion, Na-ion and Li–S batteries are prominent methods available for power storage with wearable electronics.^15^ Supercapacitors play a pivotal role among wearable energy storage devices with excellent cycle lifetime, fast charging/discharging rates, and high power density.^184^ After their discovery in 1957 by H. I. Becker, recent studies have achieved 100–150 Wh kg^–1^ energy density, 10 kW kg^–1^ power density, 90–95% energy efficiency, and superior cycling stability (>30000) without the need of chemical reactions to store energy.^101,185^

**Figure 12:**
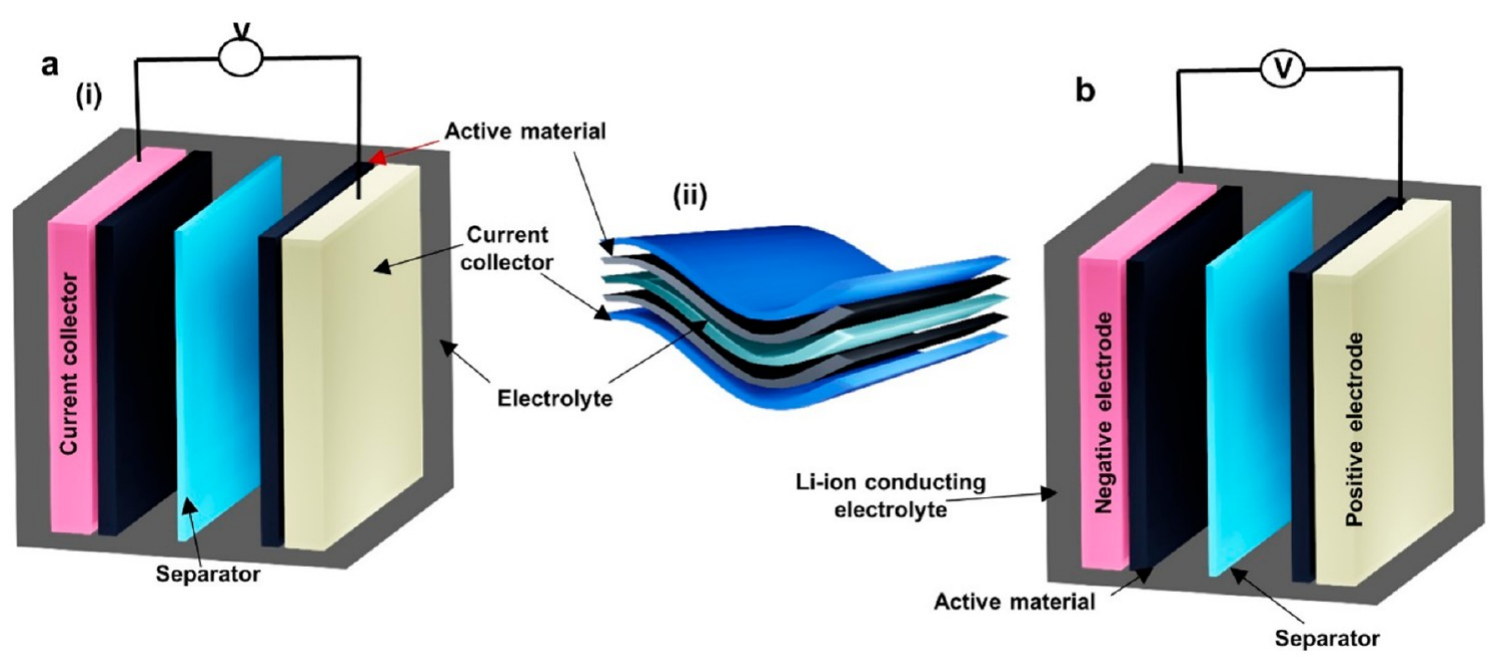
Schematic representation of wearable energy storage devices. (a) Schematic of the supercapacitor and (b) schematic of Li-ion battery. Adapted with permission under a Creative Commons [CC BY] License from ref (186). Copyright 2022 The Authors. Advanced Science published by Wiley-VCH GmbH.

Supercapacitors are currently categorized into three main techniques: electric double-layer capacitors (EDLCs), pseudocapacitors, and a hybrid of these two approaches. A typical EDLC architecture consists of a thin layer of separator sandwiched between inner electrolyte-coated carbon form electrodes (activated carbon, CNT, and graphene^184,186^) (Figure 12a). The separator must serve as both an insulator and a conduit for electrolyte ion transfer in these devices.^187^ The energy storage mechanism of EDLC occurs when a potential difference is applied between the electrodes, creating double-layer electrostatic charging at the electrode/electrolyte interface without Faradaic reaction.^66^ In contrast to an EDLC, pseudocapacitors have used metal oxides such as MnO_2_, V_2_O_5_, and RuO_2_ or conductive polymers such as PANI, PP, and PEDOT.^186,188^ With these materials, a rapid Faradaic reaction is prominent in the electrode and electrolyte interface (charging, electrode reduction with adsorption cations from the electrolyte; discharging, reverse process) in pseudocapacitors, resulting in higher energy density and reduction in cycle life than an EDLC.^186^

In addition, flexible Li-ion batteries are prominent among storage devices for wearable applications due to scalability, mechanical robustness, and electrochemical sustainability.^65,101^ In 1991, Sony and Kasei developed the commercial lithium-ion batteries. The architecture of Li-ion batteries contains Li-based cathodes, such as lithium titanate oxide (LTO)/lithium iron phosphate or LTO/lithium manganese oxide. The anode typically contains carbonaceous materials and electrolytes (Figure 12b). Other than traditional materials, PEDOT:PSS, polydopamine, PP and carbon nanofiller reinforced cellulose, PLA, PVDF, and PVA are notable in Li-ion and supercapacitor applications.^101^ However, the performance of supercapacitors and Li-ion batteries noticeably depends on the porosity and surface properties of electrode materials.^184^ Interestingly, most of these materials are electrospinnable polymers and provide high surface area, porosity, and flexibility required by each device.

Ariyamparambil and Kandasubramanian have explained that electrospinning can enhance the porosity and specific surface area of metal oxide polymers aimed at flexible electrodes for supercapacitor applications.^189^ In addition, Prasannakumar et al. have provided a comprehensive review on the importance of using electrospinning for tuning conductive polymers for supercapacitor application.^52^ It was demonstrated that having a porous structure due to the electrospinning technique affects factors such as enhancement of capacitance and provides high contact between the conductive layer and electrolyte. This also enables the insertion of electrolyte material which boosts electrochemical performance, increasing mass loading and producing binder-free flexible electrodes for supercapacitor applications. Conjugated polymers, namely conductive polymers such as PANI, PP, and PEDOT, are widely electrospinnable that may be employed as conductive layers for supercapacitors and batteries.^52^

**Figure 13:**
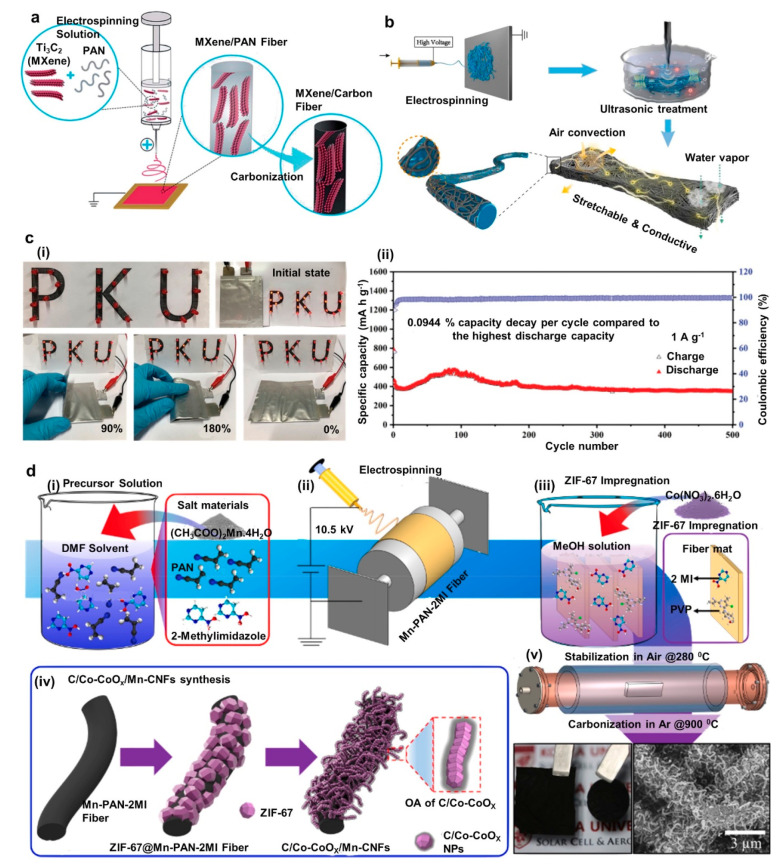
Electrospinning modified wearable energy storage devices. (a) Schematic illustration of producing Ti_3_C_2_T_*x*_ MXene/PAN nanofibers using electrospinning. Reprinted from ref (190) with permission. Copyright 2019 Royal Society of Chemistry. (b) Schematic of stretchable PU/CNT electrode fabrication using electrospinning followed by an ultrasonic cavitation process. Reprinted from ref (191) with permission. Copyright 2022 Elsevier BV. (c) V_2_O_3_ electrospun electrode based Li-ion battery performance: qualitative performance under different deformation (i) and cycling performance up to 500 cycles (ii). Reprinted from ref (192) with permission. Copyright 2020 Wiley-VCH GmbH. (d) Schematic showing of electrospinning and post processing for a ZIF-67-loaded Mn-PAN-2MI-based supercapacitor: electrospinning precursor (i), schematic of process (ii), ZIF-67 wet impregnation process over electrospun sample (iii), transformation process up to C/Co-CoO_*x*_ nanotubes (iv), and stabilization and carbonization (upper) and SEM (lower) of decorated samples (v). Reprinted from ref (193) with permission. Copyright 2023 Elsevier BV.

Binder-free electrodes can produce higher areal capacitance for supercapacitor applications. This has the added advantage of increasing the flexibility and breathability required in wearable applications. Adding Ti_3_C_2_T_*x*_ MXene into a CNF network can enhance the electrochemical activities to improve the performance as electrodes for supercapacitors. Levitt et al. successfully electrospun Ti_3_C_2_T_*x*_ MXene, and PAN (MXene to PAN 2:1) to produce such electrodes (Figure 13a). Subsequently, electrospun nanofibers were carbonized to produce the CNF, resulting in an aeral capacitance of 205 mF cm^–2^ at 50 mV s^–1^. Compared with pure CNF, the authors reported nearly 3 times improvement of the capacitance.^190^ In another example, Luo et al. have embedded long single-walled CNT into an electrospun PU nanofiber mat using high-power ultrasonic cavitation targeting highly stretchable electrodes (Figure 13b). The developed electrode was thin and flexible with a thickness of 50–200 μm and recoverable stretching of up to 200% and stability of up to 20000 bending cycles. Breathability was good with an air permeability of 22.83 mm s^–1^ (at a pressure difference of 100 Pa) and water vapor transmission of 0.008 g cm^2^ h^–1^. Reported electrical properties included a sheet resistance of 30–50 Ω sq^–1^, and the device had considerable washing durability. In addition, a supercapacitor was developed by depositing PANI on this electrode. This resulted in a specific capacitance of 543 F g^–1^ at a current density of 1 A g^–1^, and 83% of this was retained after 20%, 200 stretching cycles. The fully charged wearable supercapacitor was demonstrated to light a commercial red LED (1.5 V) for 30 s.^191^

Traditional electrodes used in Li-ion batteries tend to break or peel off from the current collector after several bending cycles due to low flexibility and stretchability, resulting in rapid capacity decay during high mass loading. Zhang et al. have fabricated freestanding V_2_O_3_ with multichannel CNF using an electrospinning technique to produce an anode electrode for wearable Li-ion battery application (Figure 13c). Due to their binder-free nature, these electrodes inherently possess high energy density and low weight. The Brunauer–Emmett–Teller method confirmed that the electrode had a large surface area of 455.136 m^2^ g^–1^. Notably, the electrode could exhibit a specific capacity of 487.7 mAh g^–1^ at 5 A g^–1^ even after 5000 cycles (0.00323% decay rate). The final device manufactured with the developed electrode has minimally varying outputs under different deformations. It retains a specific capacity of 348.3 mAh g^–1^ at 1 A g^–1^ upon 500 cycles (Figure 13c (ii)).^192^

Electrospinning is a versatile method to improve the connection between liquid electrolytes attached to supercapacitor applications. This is important to ensure reliable connectivity in wearable applications, particularly when the textile will be subjected to movement during wear. Recently, More et al. used electrospinning to produce nanofibers with PAN-2-methyl imidazole (PAN-2MI) and optimized Mn concentration to acquire high conductivity suitable for such applications. After that, zeolitic imidazolate frameworks (ZIF)-67 were loaded into the nanofiber network along with PVP using wet impregnation for high energy storage and better electrochemical stability (Figure 13d (i–iii)). The developed samples have carbonized to produce C/Co-CoO_*x*_ nanotubes suitable for the application (Figure 13d (iv,v)). The final device was made with KOH as the electrolyte and Celgard 3501 as the separator and could exhibit a capacitance of 1263 mF cm^–2^. Furthermore, the device could deliver a power density of 2.8 mW cm^–2^ and an energy density of 0.32 mWh cm^–2^ with a potential window of 0–1.4 V. Ultimately it was demonstrated that the system was capable of powering three commercial LEDs for 23 min.^193^

### 3.6 Electrospinning-Enabled Communication Devices: Wearable Antennas

Antennas serve as the key enabling technology for connectivity in wearable wireless electronics. It enables the transmission and reception of electromagnetic waves, facilitating wireless communication between the wearable device and other devices or networks. The antenna performance and design directly impact the reliability, range, data transfer rates, and overall functionality of the wearable device’s wireless connectivity.

The human body can be used as an operating environment for an antenna. In addition to its lossy nature, it is extremely dynamic, necessitating specific physical requirements that may or may not be required for other applications. Wearable antennas are desired to be lightweight and compact in size in order to fit within the limited space typically available in wearable electronics. When size is not a constraint, the antenna must be conformal, flexible, or stretchable, to be able to adapt to the irregular contours and movements of the human body. There is also a need to make the antenna visually unnoticeable by making it optically transparent or easily integrated into daily attire. All of these features are essential to provide a more immersive and comfortable experience for the wearers in long-term use. When the antenna is in direct contact with the human body, it is also important to ensure the antenna’s permeability and biocompatibility to ensure safety for prolonged skin contact, i.e., minimizing the risk of skin irritation, allergies, and other adverse effects. While considering the aforementioned physical characteristics, the antenna designer must strive for optimum and consistent antenna performance regardless of human body proximity and movement, particularly in terms of antenna efficiency and input impedance matching. This is necessary to ensure reliable and high-quality wireless connectivity while minimizing power consumption.

Significant efforts have been devoted to the use of unconventional materials and fabrication techniques in order to accomplish the aforementioned qualities, which may not be met by traditional printed circuit board (PCB) based antennas.^194,195^ Electrospinning began to emerge as one of the promising methods for fabricating flexible wearable antennas. Electrospinning enables the production of nanofibers that are inherently flexible and porous and have a high ratio of surface area to volume, making them suitable for the realization of flexible, lightweight, and breathable antennas. Importantly, electrospinning facilitates the use of different material compositions on a wide range of substrate materials with precise control over the size and arrangement of the produced nanofibers. This allows electrospinning to be employed to fabricate various parts of the antenna (i.e., conductive and/or nonconductive parts) with a certain level of flexibility in customizing their resultant electrical and mechanical properties. Two examples of wearable antennas created using the electrospinning method are provided below.

**Figure 14:**
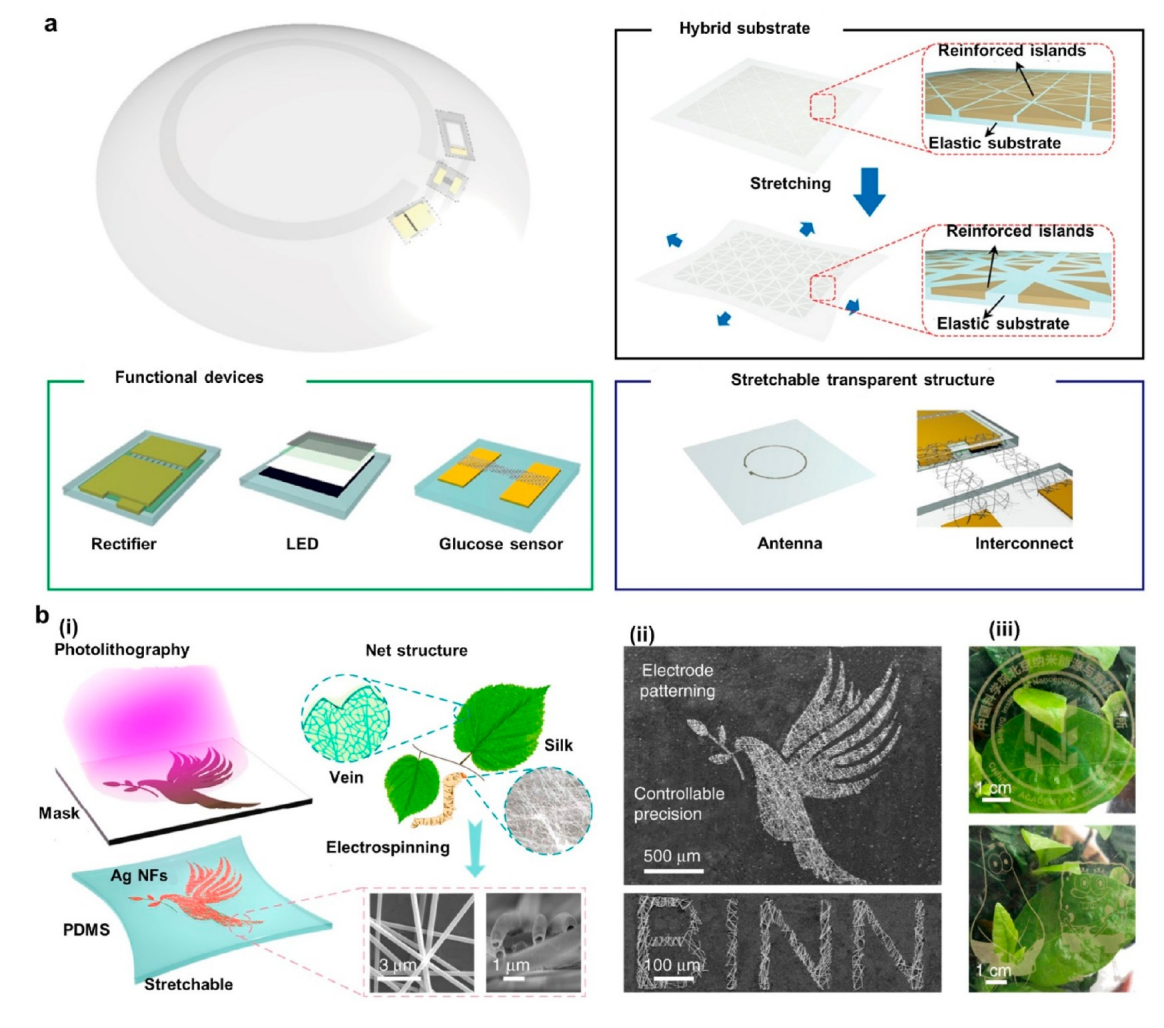
Wearable electrospun antenna applications. (a) Schematic of smart contact lens for glucose monitoring using electrospun antenna, hybrid substrate, functional devices and stretchable transparent structure. Reprinted from ref (196) with permission. Copyright 2018 The Authors, some rights reserved; exclusive licensee AAAS. Distributed under a CC BY-NC 4.0 license http://creativecommons.org/licenses/by-nc/4.0/. Reprinted with permission from AAAS. (b) Epidermal radio frequency antenna development inspiring natural structure depicted: development flowchart (i), SEM images of developed samples (ii), and actual images after fabrication (iii). Reprinted with permission under a Creative Commons [CC BY] License from ref (18). Copyright 2020 The Authors. Published by Springer Nature.

Electrospinning was utilized by Park et al.^196^ to create a stretchable and transparent loop antenna, which was used to wirelessly power a smart contact lens for glucose monitoring (Figure 14a). On the target substrate, a suspension of Ag nanoparticle ink in ethylene glycol was electrospun to form ultralong Ag nanofibers. Upon annealing at 150 °C for 30 min, the produced Ag nanofibers (maximum thickness of 2 μm and average diameter of 338 + 35 nm) were patterned into a single-loop structure (diameter of 12 mm and trace width of 5 mm) through photolithography and wet etching. Depending on the electrospinning parameters, the constructed Ag nanofibers can exhibit a sheet resistance and transparency in the range of 1.3 Ω sq^–1^ with 90% transparency to 0.3 Ω sq^–1^ with 72% transparency. In addition, the nanofibers also demonstrated an exceptional stretchability of up to 30% in tensile strain. At a distance of 5 mm from a transmitting coil, an integrated rectenna attained a power transmission efficiency of 21.5% at 50 MHz.

In another study,^18^ Zhang et al. demonstrated highly stretchable and transparent antennas for power transfer and information identification. PVA nanofibers were created using electrospinning, followed by the application of a thin layer of silver through magnetron sputtering. Photolithography and wet etching were then applied to form the nanofibers into the target shapes. The effect of different fabrication parameters (e.g., Ag NF densities, electrospinning durations, and orientations of the NFs) on the electrical properties of the developed coils (i.e., inductance, sheet resistance, and quality factor) was investigated in depth (Figure 14b). This was followed by research into the impact of number of turns and repetitive strain on the aforementioned electrical properties and, ultimately, power transfer efficiency. The authors demonstrated a five-turn coil with an efficiency level of 15% (decreasing from 35%) at 10 MHz under a severe tensile strain of 100% and a transmission distance of 2 cm. Increased parasitic capacitances and decreased conductivity due to fractures/cracks on nanofibers under high tension were identified as the cause of the efficiency drop. Moreover, the authors successfully demonstrated numerous complex functional wireless electronics employing near-field communication and frequency modulation technology for content recognition and long-distance transmission (>1 m).

It is important to note that while electrospinning offers several advantages for wearable antenna design, it also poses some challenges as implied by the above examples. Developing an antenna through the electrospinning process is typically not as straightforward as additive manufacturing techniques such as inkjet or screen printing. Typically, further processing stages are required, such as patterning the nanofibers into the desired shapes and assembling them into an antenna. In addition, the random nature of nanofibers may raise concerns about their reproducibility.

## 4 Possible Integration Methods with Wearable Electronics and Factors to Consider

Conventional plastic substrate based wearable electronic devices are rigid and bulky, making them uncomfortable to wear for a long time. Typically, developments have focused solely on improving power generation, storage, and communication aspects rather than mechanical and aesthetic performances. Considering all these factors, the use of electrospinning along with traditional textile engineering concepts can fulfill the requirements of performance optimization as well as improving wearable characteristics.^1,66^ Electrospinning-based self-powered communication systems can be embedded into garments in a few different ways.^36^ This section of the review will provide the most efficient methods which can be used to incorporate these devices into garments with minimal impact on the wearer.

From a textile engineering perspective, these devices can be grouped into two main categories depending on the production stage: fiber/yarn-based systems (converted into fabrics using weaving, knitting, braiding, sewing, or embroidery techniques) and fabric-based systems which are produced by electrospun layers. In textile engineering, fiber is considered as the fundamental building block and all the mechanical and aesthetic improvements start at this stage. Conversion of fiber into yarn can be achieved through the process of spinning followed by twisting or plying. Traditionally ring spinning has been used as the primary spinning technique, and recently air jet, rotor, wrap, and friction spinning have become popular, providing additional functionalities such as extensibility, uniformness, strength, and comfortability.^197^

Self-powered wearable wireless communication systems mainly use commercial yarns that are already twisted as the core and add a functional polymer sheath using electrospinning techniques.^1,36,51,169,198^ As an example, Dai et al. created a piezoelectric yarn by electrospinning P(VDF-TRFE) onto a copper wire. This yarn was then used as the weft and warp yarn in woven fabric, resulting in a final product that exhibited exceptional gas permeability (1041.4 mm/s) when compared to cotton, polyester, and wool fabrics. The device also exhibited a higher β phase, achieving a *V*_OC_ of 2.7 V and an *I*_SC_ of 38 nA under 15 N force. Additionally, this electrospun sheath based yarn offered excellent drapability and sufficient tensile properties, making it ideal for practical use.^199^

In contrast, some recent developments have focused on using electrospinning to directly produce yarns targeting wearable electronic applications.^200,201^ Nan et al. have developed highly stretchable and conductive nanofiber yarn by double conjugate electrospinning technique. By inverting and tapering graphene oxide-doped PAN electrospun fibers into a hollow nanoweb on a funnel and then twisting them, a yarn was formed. To increase conductivity, the yarn was coated with PP using *in situ* polymerization, resulting in an increase from 94.37 S cm^–1^ to 10.5 S cm^–1^. The electrospinning technique not only increased pressure sensitivity by increasing (gauge factor of 4.08) contact points and cumulative contact area, but also allowed the yarn to detect strains from 0.1% to 100% and repeatable up to 10,000 cycles with minimal deterioration.^200^

Targeting specific postfabric manufacturing techniques, it is essential to maintain sufficient twist or plying, yarn thickness, yarn tenacity (breaking load as a fraction of unit length), stretchability and length. In weaving and knitting there is always a minimum length required to produce a fabric. Some of these parameters have been highlighted in our previous publications.^1^ For instance, Zhi et al. highlighted that twist per meter (TPM) (= has an impact on the geometry of fabric manufacturing as well as the β phase formation of particular materials for higher output generation in energy harvesting and self-powered sensing applications.^36,202^

Weaving is a process of manufacturing fabrics by interlacing two sets of yarns, known as warp and weft, at the right angle. Plain, twill, sateen, and satin are prominent weaving structures in textile engineering. Traditionally, air jet, water jet, rapier, and projectile machines produce such structures with single-phase or multiphase techniques. Recently, 3D weaving techniques have gained attention to produce highly mechanically stable fabrics with different functionalities.^203^ When there is a requirement to interlace two types of functional yarns to produce the final device for wearable electronic application, weaving can be used as a prominent technique due to ease of fabrication and cost effectiveness.^1,48,165,204^ Furthermore, the interlacing points and crimp can be controlled using weaving structures such as plain and twill. Based on the application, the active surface area can be increased or decreased using satin and sateen structures.^1,202^ Unless the yarn is coated with a secured material, based on the delaminating nature of electrospinning sheath-based yarns, using it as the weft yarns instead of warp yarns (during weaving warp yarns are going through high tension and friction which could damage the electrospun coating) while manufacturing woven fabrics would be advisible.

In contrast, the knitting technique uses a single yarn with interloping to produce a warp- or weft-knitted fabric. Structurally, weft-knitted fabrics are highly extensible in one direction, while warp-knitted fabrics are mostly balanced in both directions.^205^ Compared with woven fabrics, knitted fabrics can take the human body’s shape, making it a more suitable technique for manufacturing intelligent garments. Specifically, mechanical self-powered sensors can be closely embedded into the targeted area using knitting techniques with advanced electrospinning-modified yarns. In addition, the rib knitting structure has higher stretchability, making it suitable for energy-harvesting applications.^206^ Furthermore, using techniques such as intarsia and seamless knitting, the devices can be localized in the structure with minimal impact on the wearable and aesthetic performance of the fabric.^207,208^ Due to high frictional force in circular and flatbed higher gauge (number of needles per inch in the knitting machine), it can lead to delamination of directly electrospun yarns without a binder. Hand knitting or lower gauge flatbed knitting^1^ techniques are more suitable for electrospinning yarn-based devices. Moreover, overcoming the delamination nature (using a suitable binder) of electrospun yarns by producing the complete yarn with electrospinning will lead to high-speed seamless knitting to produce these types of intelligent garments.

Contrary to knitting and weaving, sewing and embroidery techniques can finely localize complete functioning yarns made with an electrospinning process based on the applications of energy harvesting, storage, or communication. In addition, the higher design capability of the embroidery technique makes it more suitable for producing wearable communication devices.^17^ Compared with weaving and knitting, these techniques require high tenacity for the yarns which are subjected to rigorous motions during the fabrication process. Furthermore, if the sheath is produced solely by electrospinning it may be challenging while traveling through the needle heads. If twist exists after developing the yarn, then the twist direction (*Z* direction for single- and double-needle lock stitch) is an important parameter for sewing to prevent snarling and kinking while preparing the functional device.^209^

The electrospun layer can be used as the functional or passive layer (substrate for specific functional material fabrication) based on the application. Due to factors such as ease of fabrication, ease of characterization, and fewer post-treatment processes, the use of the traditional plate-, drum-, or conveyor-based electrospinning arrangements enable layer preparation techniques for fabric-based systems. Sun et al. have demonstrated that MEGs can be created using polymeric materials, including PVA, ethyl cellulose, silk fibroin, and poly(ethylene oxide). Their electrospun fabric-based system yielded superior results compared to counter casted films of the same materials, with poly(ethylene oxide) achieving up to 0.83 V. By adjusting the thickness, pore size, and surface area of the fabric, output can be further enhanced by increasing the absorption gradient between electrodes.^210^ All these examples discussed above in the previous sections have used either material selection or postfabrication chemical treatments to optimize the results. Specifically, in TENG, PENG, and SEHG developments, optimizing the thickness parameters to increase the sensitivity or power outputs is essential. Furthermore, facial fabrication techniques (fabricating two functional materials into the same electrospun sample front and back side) make them suitable for developing electrodes and functional material in one step. Electrospun layer-based systems are mostly required to comply with traditional woven or knitted fabric substrates in order to maintain the required smart functionalities.^202^

From an application perspective, mechanical properties are vital to electrospun yarns or membranes. In this review, we have covered instances where mechanical properties have been optimized using techniques such as changing the orientation of the electrospinning layer, changing the thickness of the layer, changing material processing parameters, and changing the diameter of the fibers. Rashid et al. thoroughly examined the relationship between mechanical properties and application of electrospun materials.^54^ To improve these properties based on the end application’s requirements, various techniques can be used, such as adding inorganic or organic fillers. For instance, MWCNT can be added to poly(l-lactic acid),^211^ or interwind nanofiber matrices can be created from polymer blends, such as adding TBAB to CA and creating a tree-like structure with TBAB branches.^180^ Polymer structures can also be modified, such as electrospun PAN peroxidation and copolymerization.^212^ Han et al. have also demonstrated that post-treatments like annealing, stretching, twisting, solvent steam treatment, postcompounding, and cross-linking can enhance the mechanical properties of electrospun membranes.^213^

## 5 Testing and Validation Techniques

The scaling and commercialization of electrospinning-based self-powered wireless communication systems must be thoroughly investigated. Based on technology readiness levels (TRL), most of the devices are either in level 3 (applied research and/or laboratory test completed) or level 4 (small-scale prototype ready in a laboratory environment).^214^ Therefore, most prototype developments must be improved with standard test procedures required for accepting these devices as commercially viable products. In textile testing, main bodies such as ASTM (American Society for Testing and Materials), BSI (British Standard Institution), ISO (International Organization for Standardization), and AATCC (American Association of Textile Chemists and Colorists) are responsible for developing standard procedures.^1^ Furthermore, International Electrotechnical Commission (IEC) and Institute of Printed Circuits (IPC) provide some additional standards related to wearable E-textiles.^215^ Following these standards to validate properties such as safety, structure, comfort, durability, and aesthetics will provide more opportunities for future steps in TRLs.

Shak Sadi and Kumpikaite have provided a comprehensive review of standard testing procedures on durability testing, namely, stability and washability related to wearable applications. Interestingly, there is positive evidence for using some testing procedures to measure the washability and stability performance of wearable electrospinning-based sensors.^215−218^ In most cases, stability testing was done based on repeating performance for several cycles, while washability was performed in a container/beaker by stirring/ultrasonication. Therefore, it is recommended to follow standard procedures such as AATCC 61-2006, ISO 6330 A7, or AATCC 135 to test the washability of functional devices for end-user reliability. In addition, AATCC TM 210 (evaluation of resistance before and after exposure to some conditions), IEC 63203-406-1 (measure surface temperature, especially wrist-worn wearable sensors), IPC 8921 A (specifications for wearable electronics with conductive yarn based woven knitted and braided fabrics), and IPC 8981 (quality and reliability related assessment) are some of the more recently developed testing standards for wearable sensors.^215^

Humidity and temperature conditions can have an adverse impact on the performance of certain electrospinning-based devices.^219^ Surface coating or nanomaterial fabrication over electrospinning substrates can favorably improve the performance of such devices to use with variable environmental conditions.^215^ In addition, traditional testing such as tensile strength (ISO 13934-1:2013, AS 4878.6-2001), air permeability (ISO 9237:1995), elongation properties (ISO 13934-1:2013, ASTM D 5035-11(2019)), flammability (BS 5438, ISO 6941:2003) and thermal comfort (ASTM D7140/D7140M-22) can be used to measure the performance of functional fabrics.^1^ Some materials used in these sensors or modules have been restricted or limited to wearable applications. The review provided by Patra and Pariti on restricted and limited substances related to fabrics and wearable applications provides a complete insight for researchers to select a wider variety of materials for possible scalable applications^220^ (see Supplementary Note 2). For example, electrospinning solvents such as DMF, DMM, and certain acids must be fully evaporated and ensure that devices are free of those substances to use for the purpose of wearable applications.

## 6 Summary and Outlook

Increasing demand for wearable devices—smart garments have the advantage of interfacing directly with the body and its environment, and there are many applications where this can improve quality of life, citizen health, worker safety, and user experience. To make such smart garments sustainable, there is a need for energy-autonomous sensing to be integrated within the textile structure, and the development of such systems must ensure wearable properties of the textile are maintained, including comfort, flexibility, and breathability. While advances in material science, flexible electronics, and advanced manufacturing have improved the uptake of wearable technologies, the challenges of textile integration bring the need to incorporate textile engineering perspectives and evaluation of suitable textile manufacturing techniques that may be modified to create smart textiles with autonomous energy harvesting capabilities. One technique that shows great promise in achieving these goals is electrospinning, which has been extensively studied for over 70 years.^221^ This method offers a sophisticated way to fabricate sustainable materials that are soft and flexible, even when incorporating materials like CNT^222^ and metal nanoparticles.^223^ Electrospinning also enables the creation of naturally occurring entangled porous nanofiber structures with manageable transparency, which is critical for balancing performance and wearability in future wearable electronics.^224^ The potential applications for electrospun nanofibers are vast, from tissue engineering^225^ to filtration^226^ to wearable technology.^105,227^ A high surface area to volume ratio can be beneficial for TENG devices, as it can boost their charge density and overall output. Additionally, the voltage applied between the needle tip and collector in this mechanism can help with the chemical structure arrangement of most PENG materials, leading to fewer postpoling requirements compared to other film-making techniques.^228^ This approach also allows for a faster charge–discharge rate, greater energy storage capacity for storage devices, and improved transparency for wearable antenna applications, which we have discussed throughout this review paper.

Energy-autonomous wearable devices are made up of different components, including sensing, energy harvesting, wireless communications, and energy storage. Applying different electrospinning approaches can address the requirements of each of these individual components that must be compatible to fit together within a textile structure. Moreover, aside from electrical characteristics electrospinning methods have the advantage of adding functionality at the fiber or yarn level which maintains inherent breathability, permeability, and flexibility of the textile structure.

Electrospinning has been demonstrated to create mechanical energy harvesting devices based on triboelectric and piezoelectric principles. TENG and PENG devices have been deployed as both energy harvesters and self-powered sensors. Researchers have characterized them in terms of electrical performance (e.g.: *V*_OC_, *I*_SC_, charge density, sensitivity, and maximum power). Their performance has constituted the feasibility of such technologies for wearable applications, particularly for health monitoring, and a number of real-world applications have been implemented, including gait and heart rate analysis.

Aside from body movements as sources of renewable energy, solar radiation may also provide a source of energy for wearable devices. The components for flexible solar energy harvesting devices materials need to be highly conductive and efficient, with the added complexity of needing transparency for one of the electrodes to facilitate the transmission of light within the active layers. Transparent conductive inks tend to be brittle in nature, which makes flexibility and bending requirements challenging to fulfill. Configurations used in traditional photovoltaics need to be redesigned in order to meet the practicalities of real-world wearable devices; e.g., liquid electrolytes are undesirable due to potential leakage due to motion and compression. A number of approaches to overcome such challenges, including solid-state or quasi-solid-state electrolytes development have been discussed in section 3.3, which demonstrate the potential of electrospinning technique for solar yarns and fabrics that could be integrated into future smart garments.

Textiles have been developed for energy harvesting from different ambient sources around the wearer; however, the energy may be transient, and the power provided is instantaneous. In order to ensure continuous operation, energy storage components are essential to any energy autonomous wearable system. In section 3.5 we discuss the advantages electrospinning can offer to the development of different layers of energy storage components such as supercapacitors and flexible rechargeable batteries. Electrospinning can be used to create highly stretchable electrodes that are thin and flexible and can also be used to improve the connectivity between layered devices to ensure reliability and durability after repeated bending and flexing. Some of the latest developments were able to power commercial devices such as a thermo-hygrometer, watches, or calculators continuously without needing a battery.^229,230^

The greatest advantage of wearable technology is to glean physiological signals from the body in a natural way during daily life. In order to facilitate these, wireless communications are essential to transmit the relevant data to external devices such as a smartwatch, network, or relevant application. Wireless communications combined with energy harvesting and storage enables sensors to be located in body locations such as the eye which are difficult to access with sensing devices. Wearable antennas must conform to body contours and along with biocompatibility must be flexible, lightweight, and breathable, which is why electrospinning has been used to develop nanofibers that are inherently flexible and porous and have a high ratio of surface area to volume. Examples of wearable antennas are discussed in section 3.6, although the current drawback of this approach is that a number of processing stages are required to create the devices. The scalability of all the approaches discussed in this paper is an area which must be addressed in order for this technology to become commercially feasible/textile integration, and the cooperation with textile manufacturing techniques is key to furthering such innovations. This is essential for the scalability of smart textile manufacturing and ensuring that future wearable devices are compatible with textile manufacturing processes. Another key area to ensure the attributes of smart textiles is to establish testing procedures and protocols to validate durability, washability along with mechanical and electrical characteristics. A number of relevant test procedures are discussed in section 5.

In general, electrospinning has made a significant contribution to the development of wireless communication systems that can be worn comfortably. However, as mentioned earlier, these devices still need to improve in the TRL aspect. To achieve this, several challenges must be addressed, including the precise control of electrospinning parameters, maintenance of the repeatability of the process, and increase of the production speed of electrospinning nanofibers. Electrical properties are evidenced and can be further optimized by process parameters, materials, and advanced approaches. The diverse configurations that are possible with electrospinning to add functionality at different stages of textile development, directly producing fibers and yarn that can be used to weave or knit textiles or modifying fabric surfaces with functionalized layers of electrospun fibers. Integrating technology at this fundamental level is vital for future smart garments which are fit for the purpose. Another major concern is the use of harmful organic solvents, which could negatively impact the future sustainability of this technology. To overcome this challenge, Lv et al. have proposed the concept of green electrospinning, which requires further research to fully realize its potential for sustainable material processing.^231^ Additionally, maintaining a balance between wearable electronic performance and mechanical properties during the electrospinning process can be difficult. To increase production speed, techniques like needleless electrospinning, wet electrospinning, and blow electrospinning need to be explored. While polymer material processing has its advantages, improving the conductivity along with durability of the conductive material after electrospinning remains an ongoing challenge.^232^

Current challenges of scalability call for further interdisciplinary research across spheres including textile engineering, manufacturers, material science, and electronic and mechanical engineering. Sustainability must also be core to the design and consider the use of biodegradable materials where possible and consider the full product lifecycle to reduce future waste. The feasibility of deployment in larger scale studies and evaluation of the impact of wearable sensing need strong links with expertise in data analytics. Wearable sensors have the potential to generate vast quantities of data regarding population and environmental health. While there is a tradeoff in accuracy of wearable devices compared to medical gold standards for home-based 24/7 monitoring, there is the potential to glean valuable health information with appropriate analytical tools such as machine learning and AI. The number of sensors and smart devices is ever increasing, and this research field has the potential to support this continuing trend in a way that is sustainable and addresses future energy needs.

## Supporting Information Available

Potential of using electrospinning on wearable energy harvesting, energy storage, and antenna applications and maximum allowable limit and testing standards for certain materials used in wearable self-powered wireless communications systems (PDF)

## Abbreviations

TENG: triboelectric nanogenerator
SEHG: solar energy harvesting
DSSC: dye-sensitized solar cells
PSC: perovskite solar cells
OSC: organic solar cells
PCE: power conversion efficiency
PVDF: poly(vinylidene fluoride)
PI: polyimide
PVDF-TrFE: polyvinylidene fluoride-trifluoroethylene
PVDF-HFP: poly(vinylidene fluoride-hexafluoropropylene)
EDLC: electric double-layer capacitor
PEDOT: poly(3,4-(ethylenedioxy)thiophene)
PANI: polyaniline
PAN: polyacrylonitrile
PLA: poly lactic acid
PP: polypyrrole
CNT: carbon Nanotube
*V*_OC_: open circuit voltage
*I*_SC_: short circuit current
DDEF: distance-dependent electric field
PDMS: polydimethylsiloxane
DMF: dimethylformamide
PA: polyamide
CA: cellulose acetate
PU: polyurethane
*F*(β): percentage of β phase
TEG: thermoelectric energy generator
EEG: evaporative energy generator
PCB: printed circuit board
TRL: technology readiness level

## Vocabulary

electrospinning: technique that uses applied voltage to create nanometer-scale polymer fibers from polymer solutions or melts
energy harvesting: the process of transforming the energy available in the surrounding environment into electrical energy
wearable electronics: category of electronic devices that can be worn as accessories embedded in clothing, or even implanted in the body
technology readiness level: measurement of the maturity level of a technology
fiber: fundamental building unit of a textile that can be spun into yarn and made into fabrics
