# Supplementary material for: The Potential of Electrospinning to Enable the Realization of Energy-Autonomous Wearable Sensing Systems
Source: ACS Nano. 2024 Jan 17;18(4):2649–84. doi: 10.1021/acsnano.3c09077 (PMC10832067; doi:10.1021/acsnano.3c09077)
Supplement: Supplementary file 1 — nn3c09077_si_001.pdf [file nn3c09077_si_001.pdf]

The potential of electrospinning to enable the realisation of energy-autonomous wearable sensing systems.

K.R. Sanjaya Dinuwan Gunawardhana[1,2], Roy B. V. B. Simorangkir[3], Garrett Brian McGuinness[4], M. Salauddin Rasel[2], Luz A. Magre Colorado[1], Sonal S. Baberwal[1], Tomás E. Ward[2,5], Brendan O'Flynn[3], Shirley M. Coyle[1,2]*

[1]School of Electronic Engineering, Dublin City University, Glasnevin, D09Y074, Dublin, Ireland

[2]Insight SFI Centre for Data Analytics, Dublin City University, Glasnevin, D09Y074, Dublin, Ireland

[3]Tyndall National Institute, Lee Maltings Complex Dyke Parade, T12R5CP, Cork, Ireland

[4]School of Mechanical Engineering, Dublin City University, Glasnevin, D09Y074, Dublin, Ireland

[5]School of Computing, Dublin City University, Glasnevin, D09Y074, Dublin, Ireland

Supplementary Document

Supplementary note 1: Potential of using electrospinning on wearable energy harvesting, energy storage and antenna applications.

Material	Electrospinning Parameters	Functional performance	Mechanical /Wearable performance	Final integration method to garments	Year
**TENG applications**					
Electrospun PVDF – negative	PVDF 10 wt% DMF: Acetone (3:2), Needle gauge-23G, Flow rate 1 mL/h, Voltage – 15 kV, TCD – 8	VOC -1036 V, ISC – 196 μA, power density – 14.8 W m-2 through 6 MΩ (checked 45 x 45	After 10 washes no degradation of output (detergent 1 g/l 20 min	Attached into conductive fabric and	2019 [1]

Natural rubber – positive	cm, Collection with rotary collector 2800 rpm	mm2 56N, 6 Hz), can lit 595 commercial LED, charge 47 µF in 16 seconds	washing + 10 min rinsing twice)	sewn in 3 pieces of cloth under arm	
Electrospun PVA/Mxene – negative<br><br>Electrospun Silk fabrion – positive	10 (w/v)% PVA in DI water + 30 (v/v)% Mxene, needle 0.68 mm inner diameter, Flow rate – 18 µl/min, Voltage – 18 kV, TCD – 11 cm, flat collector	VOC -118.4 V, maximum power – ~1087.6 mW m-2 through 5 MΩ (checked 10N, 10 Hz), can lit 38 commercial LED, charge 22 µF up to 2.9 V, durable for 124000 cycles	Not specified	As a membrane itself on fingers, knee and neck	2019 [2]

	7.5 % Silk fibrion in HFIP, needle 0.58 mm inner diameter, Flow rate – 20 µl/min, Voltage – 20 kV, TCD – 12 cm, flat collector				
Electrospun PVDF-HFP/ styrene- ethylene-	PVDF-HFP 12 wt% DMF: Acetone (3:2), Flow rate 2.1 mL/h, Voltage – 20 kV, TCD – 15 cm	VOC -85 V, ISC – 4 µA, Charge – 15 nC at 0% strain, power density – 219.66 mW m-2 through 20 MΩ (checked 20 x	Stretchability-~490%, great hydrophobicity (static water contact angle of - ~1400) and	Attached as membrane to finger.	2020 [3]

butylene-styrene (SEBS) – negative<br><br>Skin or aluminum-positive	SEBS electro-spraying, 0.9 mL/h	20 mm2 30N, 5 Hz) can lit 200 commercial LED, and use to power electronic watch	breathability (71% of cotton).		
Electrospun Polycaprolactam -positive	Polycaprolactam 20 wt% methane acid: ethanoic acid (4:1), Flow rate 0.1 mL/h, Voltage – 20 kV, TCD –	VOC -270 V, ISC –11 μA, Charge – 55 nC, power density – 25 W m-2 through 50 MΩ	Stretchability – 400 %,	Integrated into fitness brace	2020 [4]

Ecoflex 00-30 - negative	20 cm, Collection with rotary collector 2800 rpm<br><br>SEBS electro-spraying, 0.9 mL/h	(checked 15 x 15 mm2 frequency (3.5 Hz) pressure - ~200 kPa), can charge 2.2 µF, ~ 85 % performance after 5000 cycles			
Electrospun Ethyl cellulose/Polya	EC (40 w/v%), PA 6 (25 wt %) in formic and acetic acid mixture, Needle gauge-19G, Flow rate 0.6	VOC -~50 V, ISC – ~2.5 µA, power density – 290 mW m-2 through 100 MΩ (checked 20 x	PA 6 mat breaking strength 25.3 MPa and elongation at break of 86.9%	Attached to underarm cloth to monitor hand gestures.	2021 [5]

mide 6 (EC/PA 6) – positive<br><br>Electrospun PVDF/MXene - negative	mL/h, Voltage – 22 kV, TCD – 15 cm<br><br>PVDF 15 wt% DMF: Acetone (3:2), Needle gauge-19 G, Flow rate 1 mL/h, Voltage – 18 kV, TCD – 15 cm	20 mm2 6 mm amplitude, 5 Hz), can lit 180 LED			
Electrospun PA 66 – positive	PA 66 15 wt% Formic acid, Needle gauge-18 G, Flow rate 1 mL/h,	VOC -166 V, ISC – 8.5 μA, power density – 93 mW m-2 through 10 MΩ (checked 50 x	Water vapour permeability -712 ± 16 g/m2 ·24 h	Electropsun membranes wrapped around stainless stell	2021[6]

Electrospun P(VDF-TrFE) - negative	Voltage – 25 kV, TCD – 25 cm, rotary collector at 500 rpm<br><br>P(VDF-TrFE) 20 wt% DMF: Acetone (3:7), Needle gauge-18 G, Flow rate 1 mL/h, Voltage – 25 kV, TCD – 14 cm, rotary collector at 500 rpm	50 mm2 200 N, 3 Hz), can lit 58 LED, same for 20000 cycles	Air permeability -164 ± 5 mm/s, little shrinkage after 5 washing cycles and 90 % functional performance	core and converted into woven fabric.	

Electrospun PVA – positive<br><br>Electrospun PVDF – negative<br><br>Electrospun polystyrene nanoparticles (PSC) charge	PVA in DI water, Needle gauge-19 G, Flow rate 0.8 mL/h, Voltage – 18 kV, TCD – 15 cm, rotary collector 280 rpm<br><br>PVDF 15 wt% DMF: Acetone (3:2), Needle gauge-19 G, Flow rate 0.8 mL/h, Voltage – 18 kV, TCD – 15 cm, rotary collector 280 rpm	VOC -45.1 V, ISC – 3.14 µA, power density – 58.1 mW m-2 through 60 MΩ (checked 20 x 20 mm2 , 3 Hz), can lit 183 LED, stable for 3000 cycles	Not specified	Attached into laboratory rubber glove	2022 [7]

transfer layer and a polystyrene (PS) charge-storage layer	PS/PSC 24 wt% in DMF, Needle gauge-19 G, Flow rate 1 mL/h, Voltage – 30 kV, TCD – 15 cm, rotary collector 800 rpm				
Electropsun PA 66- positive Coated PVDF - negative	PA 66 20 wt% in formic acid, Flow rate 0.01 mL/h, Voltage – 27 kV, TCD – 10 cm, rotary collector 280 rpm	VOC -100 V, current density– 24.5 mA m-2, power density – 280 mW m-2 through 4 MΩ (checked 25 x 25 mm2 , 8 N, 8 Hz), can lit 40 LED, can	Not specified	PA 66 collected on silk fabric and PVDF coated on polyester fabric	2022 [8]

		generate output voltages of ~600 mV putting in human hand			
Facile Poly-L-lysine post surface modified Nylon 11 - positive	Nylon 11, 10 wt% in formic acid: dichloromethane (1:1), Needle diameter-0.6 mm, voltage – 18 kV, Flow rate – 0.5 mL/h, TCD – 10 cm, rotary collector 80 rpm	VOC - 137 V, ISC - 3.4 μA, Charge density - 26 nC, Power density - 2 Wm-2 through 70 MΩ(checked 20 x 20 mm2 , 4.5 N, 5 Hz), Sensitivity = 9 V Pa-1,	Not specified	Cu Ni coated polyester fabric as substrate and electrodes.	2023 [9]

SrTiO3-embedded Ecoflex - negative					
**PENG applications**					
MWCNTs/P(VDF-TrFE)	P (VDF-TrFE), 15 wt% in DMF: Acetone (3:2), voltage – 12-14 kV,	VOC – 18.23 V, ISC – 2.14 µA, Power density – 6.53 µW cm-2	Young's modulus (47.6 MP), elongation at break – 76.8%	Attached as a patch with flexible	2019 [10]

	Flow rate – 1 mL/h, TCD – 15 cm, rotary collector 500 rpm	through 10 MΩ, stable for 20000 cycles		electrode and plastic sheet	
barium titanate doped polyvinylidene fluoride (BTO/PVDF)	2.25 g PVDF + 68 mg BTO in DMF: Acetone (6 mL: 4 mL), voltage – 12 kV, Flow rate – 0.6 mL/h, TCD – 10 cm	Voltage - 0.38 V/N, current - 43.9 nA/N, in 0.22 N to 19.33 N (in 30 x 30 mm2)	Not specified	Al and PET attached into electrospun membrane	2021 [11]

PVDF-TrFE nanofiber mat with barium titanate (BaTiO3) – piezoelectric<br><br>MWCNT/PU electrode material	1.4 g PVDF-TrFE + 0.14 g BaTiO3 in 10 mL of DMF/<br><br>acetone (3:2), voltage – 15 kV, Flow rate – 10 µl/min, TCD – 15 cm<br><br>3 g PU pellets to 10 mL DMF/acetone (1:1), voltage – 15 kV, Flow rate – 12 µl/min, TCD – 15 cm	Voltage - ~3 V, (1 N, 1 Hz, in 20 x 25 mm2), current –<br><br>2.72 nA N−1, in 2-10 N, stable for 12000 cycles<br><br>Electropsun PU/MWCNT electrical conductivity<br><br>5.29 S m−1	water vapor permeability is<br><br>565.43 g m−2 24 h−1	PVDF-TrFE mat sandwiched between MWCNT/PU layers and attached into a glove	2022 [12]

$(Ba_{0.85}Ca_{0.15})(Ti_{0.90}Zr_{0.10})O_3$ (BCTZ) nanoparticle into a P(VDF-TRFE) electrospun network	15% P(VDF-TRFE) dissolved in DMF:acetone (7:3) – 0-30 wt% BCTZ mixed with electrospun precursor.<br><br>voltage – 15 kV, Flow rate – 1 mL/h, relative humidity – less than 30 %	Voltage - 36.5 V, Current of 1.09 μA, and maximum power density of 507.7 μW cm^{-3} through 40 MΩ resistor.<br><br>After annealing (2 h at 145 ˚C) and poling (electric field of 100 kV cm^{-1}, 1 h at 70 ˚C) had increased the performance of	-	Detect articular bending motions, carotid pulse and voice	2022[13]

		voltage and current by 6.6 and 15.7 times respectively.			
Fluorinated $BaTiO_3$ nanoparticles to electrospun PVDF nanofibers	$BaTiO_3$ prepared by sol gel technique. Initial powder was modified with PVDF powder with annealing. 2.69 g of PVDF in DMF (22.5 mL) and 5% $BaTiO_3$ dispersed stirring for 1 h	Voltage of 1.5 V at 2N.	-	Used to measure different bending states. (0, 90, 120^0) at 1 Hz frequency. Attached into insole and walking generate 0.9 V.	2022[14]

	Flow rate – 1.5 mL/h, TCD – 10 cm, Applied voltage – 20 kV, roller collector with 1000 rpm.				
PVDF/AgNP/MXene fibers	PVDF, 18 wt% in DMSO: Acetone (1:1)+AgNP/MXene, needle 20G, voltage – 14 kV, near field electrospinning with 1mm TCD, rotary collector 1900 rpm	Sensitivity - 0.31428 V Hz−1 clapping hands – 15 V, moving arms – 7.5 V, moving a finger – 4 V, and walking – 11 V	Tensile strength - 10.5 MPa, 80% strain	PVDF/AgNP/MXene fibers sandwiched between copper tape. PET and PDMS used to completely cover	2023 [15]

BTO in PVDF mat	16 wt% PVDF + 10 % BTO in DMF: Acetone (3: 2), needle inner diameter – 0.337 mm, voltage – 22 kV, Flow rate – 0.8 mL/h, TCD – 15 cm, rotatory collector 200 rpm	Sensitivity - 11.6 V/bar in 0-5 bar, stable for 12000 cycles, response time 82.7 ms	Youngs modulus - 0.18 ± 0.03 MPa, Yield strength - 0.43 ± 0.07 MPa, Tensile strength - 0.61 ± 0.06 MPa, Elongation at break - 0.79 ± 0.03 times		2023[16]

Supplementary note 2: Maximum allowable limit and testing standards for certain materials used in wearable self-powered wireless communications systems.

Material	Maximum allowable level in finished product	Potential use in textile processing	Testing standard
Acetophenone	50 ppm	-	Extraction with MeOH / GC-MS

2-Phenyl-2-Propanol	50 ppm	-	Extraction with MeOH/GC-MS
Extractable Antimony	60 ppm	Textile, leather, polymers (R.F.A.)	DIN EN ISO 105-E04 (2013)(acid sweat solution)// ISO 17294-2 (2003) or DIN EN ISO 11885 (2009)
Extractable Arsenic	25 ppm	Textile, leather, polymers (R.F.A.)	DIN EN ISO 105-E04 (2013)(acid sweat solution)// ISO 17294-2 (2003) or DIN EN ISO 11885 (2009)
Extractable Barium	1000 ppm	Textile, leather, polymers (R.F.A.)	EN 71-3

			CNS 4797-4
Total Cadmiun	Adults: 75 ppm<br><br>Children: 40 ppm	Textile, leather, polymers (R.F.A.)	EN 1122 (2001)/<br><br>ISO 17294-2 (2003) or DIN EN ISO 11885 (2009)<br><br>Total digestion /<br><br>ISO 17294-2 (2003) or DIN EN ISO 11885 (2009)
Extractable Chromium	60 ppm	Textile, leather, polymers (R.F.A.)	Textile: DIN EN ISO 105-E04 (2013) (acid sweat solution) // ICP

			Leather: 17075 (2008)
Extractable Cobalt	Adults: 4 ppm<br><br>Children: 1 ppm	Textile, leather, polymers (R.F.A.)	DIN EN ISO 105-E04 (2013)(acid sweat solution)//<br><br>ISO 17294-2 (2003) or DIN EN ISO 11885 (2009)
Extractable Copper	Adults: 50 ppm<br><br>Children: 25 ppm	Textile, leather, polymers (R.F.A.)	DIN EN ISO 105-E04 (2013)(acid sweat solution)//<br><br>ISO 17294-2 (2003) or DIN EN ISO 11885 (2009)
Total Lead	90 ppm	Textile, leather, polymers (R.F.A.)	Total digestion /

			ISO 17294-2 (2003) or DIN EN ISO 11885 (2009)
Extractable Lead	Total: 90 ppm<br><br>Adults and children: 1 ppm<br><br>Babies: 0.2 ppm	Textile, leather, polymers (R.F.A.)	DIN EN ISO 105-E04 (2013)(acid sweat solution)//<br><br>ISO 17294-2 (2003) or DIN EN ISO 11885 (2009)
Fluorene	10 ppm	Textile, leather, polymers (R.F.A.)	

Dimethylformamide (DMFa)	500 ppm	Candidate list*	
Formamide	1000 ppm	-	
Dimethylacetamide (DMAC)	1000 ppm	Candidate list*	Headspace /GC-MS or<br>Textile: Extraction with MeOH / GC-MS or LC-MS

			Plastic: 2-step Extraction with THF and MeOH / GC-MS or LC-MS
N-Methyl-2-pyrrolidone (NMP)	1000 ppm	Candidate list*	
2-methoxypropanol	-	Candidate list*	
Toluene	-	Candidate list*	Headspace /GC-MS
Methanol	-	Candidate list*	

Ethylbenzene	-	Candidate list*	
2-(2-methoxyethoxy)-ethanol	-	Candidate list*	
Methylene chloride	-	Textile, leather, polymers (R.F.A.)	
Benzene	5 ppm	Textile, leather, polymers (R.F.A.)	Headspace /GC-MS
Carbon Disulfide/Carbon	1000 ppm	-	

Tetrachloride/Chloroform/Cyclohexanone			
1,2-Dichloroethane and Tetrachloroethylene (PERC)	1000 ppm	Textile, leather, polymers (R.F.A.)	Headspace GC-MS
1,1-Dichloroethylene	1000 ppm	-	
Ethylbenzene and Toluene	1000 ppm	Candidate list*	

Trichloroethylene	1000 ppm	Textile, leather, polymers (R.F.A.)	Headspace /GC-MS

Adopted from ref [17,18]

*Candidate list comprises chemical substances that may pose a hazard, but the evidence is not conclusive. Therefore, these items are not yet included in the Manufacturing Restricted Substances List.

Reference

(1) Zhang, J. H.; Li, Y.; Du, J.; Hao, X.; Huang, H. A High-Power Wearable Triboelectric Nanogenerator Prepared from Self-Assembled Electrospun Poly(vinylidene Fluoride) Fibers with a Heart-like Structure. *J. Mater. Chem. A* **2019**, *7* (19), 11724–11733. https://doi.org/10.1039/c9ta01956a.

(2) Jiang, C.; Wu, C.; Li, X.; Yao, Y.; Lan, L.; Zhao, F.; Ye, Z.; Ying, Y.; Ping, J. All-Electrospun Flexible Triboelectric Nanogenerator Based on Metallic MXene Nanosheets. *Nano Energy* **2019**, *59* (January), 268–276. https://doi.org/10.1016/j.nanoen.2019.02.052.

(3) Li, Y.; Xiong, J.; Lv, J.; Chen, J.; Gao, D.; Zhang, X.; Lee, P. S. Mechanically Interlocked Stretchable Nanofibers for Multifunctional Wearable Triboelectric Nanogenerator. *Nano Energy* **2020**, *78* (July). https://doi.org/10.1016/j.nanoen.2020.105358.

(4) Qi, J.; Wang, A. C.; Yang, W.; Zhang, M.; Hou, C.; Zhang, Q.; Li, Y.; Wang, H. Hydrogel-Based Hierarchically Wrinkled Stretchable Nanofibrous Membrane for High Performance Wearable Triboelectric Nanogenerator. *Nano Energy* **2020**, *67* (November

2019), 104206. https://doi.org/10.1016/j.nanoen.2019.104206.

(5) Huang, J.; Hao, Y.; Zhao, M.; Li, W.; Huang, F.; Wei, Q. All-Fiber-Structured Triboelectric Nanogenerator via One-Pot Electrospinning for Self-Powered Wearable Sensors. *ACS Appl. Mater. Interfaces* **2021**, *13* (21), 24774–24784. https://doi.org/10.1021/acsami.1c03894.

(6) Guan, X.; Xu, B.; Wu, M.; Jing, T.; Yang, Y.; Gao, Y. Breathable, Washable and Wearable Woven-Structured Triboelectric Nanogenerators Utilizing Electrospun Nanofibers for Biomechanical Energy Harvesting and Self-Powered Sensing. *Nano Energy* **2021**, *80* (July 2020), 105549. https://doi.org/10.1016/j.nanoen.2020.105549.

(7) Hao, Y.; Huang, J.; Liao, S.; Chen, D.; Wei, Q. All-Electrospun Performance-Enhanced Triboelectric Nanogenerator Based on the Charge-Storage Process. *J. Mater. Sci.* **2022**, *57* (8), 5334–5345. https://doi.org/10.1007/s10853-022-06927-0.

(8) Bairagi, S.; Khandelwal, G.; Karagiorgis, X.; Gokhool, S.; Kumar, C.; Min, G.; Mulvihill, D. M. High-Performance Triboelectric Nanogenerators Based on Commercial Textiles: Electrospun Nylon 66 Nanofibers on Silk and PVDF on Polyester. *ACS Appl. Mater. Interfaces* **2022**, *14* (39), 44591–44603.

https://doi.org/10.1021/acsami.2c13092.

(9) Prasad, G.; Graham, S. A.; Yu, J. S.; Kim, H.; Lee, D.-W. W. Investigated a PLL Surface-Modified Nylon 11 Electrospun as a Highly Tribo-Positive Frictional Layer to Enhance Output Performance of Triboelectric Nanogenerators and Self-Powered Wearable Sensors. *Nano Energy* **2023**, *108* (January), 108178. https://doi.org/10.1016/j.nanoen.2023.108178.

(10) Zhao, C.; Niu, J.; Zhang, Y.; Li, C.; Hu, P. Coaxially Aligned MWCNTs Improve Performance of Electrospun P(VDF-TrFE)-Based Fibrous Membrane Applied in Wearable Piezoelectric Nanogenerator. *Compos. Part B Eng.* **2019**, *178* (June), 107447. https://doi.org/10.1016/j.compositesb.2019.107447.

(11) Su, Y.; Li, W.; Yuan, L.; Chen, C.; Pan, H.; Xie, G.; Conta, G.; Ferrier, S.; Zhao, X.; Chen, G.; Tai, H.; Jiang, Y.; Chen, J. Piezoelectric Fiber Composites with Polydopamine Interfacial Layer for Self-Powered Wearable Biomonitoring. *Nano Energy* **2021**, *89* (PA), 106321. https://doi.org/10.1016/j.nanoen.2021.106321.

(12) Luo, Y.; Zhao, L.; Luo, G.; Li, M.; Han, X.; Xia, Y.; Li, Z.; Lin, Q.; Yang, P.; Dai, L.; Niu, G.; Wang, X.; Wang, J.; Lu, D.; Jiang, Z. All Electrospun Fabrics Based

Piezoelectric Tactile Sensor. *Nanotechnology* **2022**, *33* (41). https://doi.org/10.1088/1361-6528/ac7ed5.

(13) Park, S. C.; Nam, C.; Baek, C.; Lee, M.-K. K.; Lee, G.-J. J.; Park, K.-I. Il. Enhanced Piezoelectric Performance of Composite Fibers Based on Lead-Free BCTZ Ceramics and P(VDF-TrFE) Piezopolymer for Self-Powered Wearable Sensors. *ACS Sustain. Chem. Eng.* **2022**, *10* (43), 14370–14380. https://doi.org/10.1021/acssuschemeng.2c05026.

(14) Wang, S.; Zhang, L.; Wang, L.; He, Y.; Wu, M. Fluorinated Barium Titanate Nanoparticles for Wearable Piezoelectric Power Generation. *ACS Appl. Nano Mater.* **2022**, *5* (3), 3352–3360. https://doi.org/10.1021/acsanm.1c03777.

(15) Pan, C.-T.; Dutt, K.; Kumar, A.; Kumar, R.; Chuang, C.-H.; Lo, Y.-T.; Wen, Z.-H.; Wang, C.-S.; Kuo, S.-W. PVDF/AgNP/MXene Composites-Based near-Field Electrospun Fiber with Enhanced Piezoelectric Performance for Self-Powered Wearable Sensors. *Int. J. Bioprinting* **2022**, *9* (1). https://doi.org/10.18063/ijb.v9i1.647.

(16) Li, J.; Yin, J.; Wee, M. G. V.; Chinnappan, A.; Ramakrishna, S. A Self-Powered Piezoelectric Nanofibrous Membrane as Wearable Tactile Sensor for Human Body

Motion Monitoring and Recognition. *Adv. Fiber Mater.* **2023**, *5* (4), 1417–1430. https://doi.org/10.1007/s42765-023-00282-8.

(17) Patra, A. K.; Pariti, S. R. K. Restricted Substances for Textiles. *Text. Prog.* **2022**, *54* (1), 1–101. https://doi.org/10.1080/00405167.2022.2101302.

(18) G - Star Raw. Restricted Substances List for Garments. **2016**, No. November, 1–45.
